# Role of CRP2-MRTF interaction in functions of myofibroblasts

**DOI:** 10.1247/csf.23004

**Published:** 2023-05-11

**Authors:** Ken’ichiro Hayashi, Shinri Horoiwa, Kotaro Mori, Hiroshi Miyata, Reuben Jacob Labios, Tsuyoshi Morita, Yuka Kobayashi, Chiemi Yamashiro, Fumiaki Higashijima, Takuya Yoshimoto, Kazuhiro Kimura, Yoshiaki Nakagawa

**Affiliations:** 1 Department of RNA Biology and Neuroscience, Osaka University Graduate School of Medicine, 2-2 Yamadaoka, Suita, Osaka 565-0871, Japan; 2 Division of Applied Life Sciences, Graduate School of Agriculture, Kyoto University, Kitashirakawa-Oiwakecho, Sakyo-ku, Kyoto 606-8502, Japan; 3 Department of Surgery, Osaka International Cancer Institute, 3-1-69 Otemae, Chuo-ku, Osaka 541-8567, Japan; 4 Department of Biology, Wakayama Medical University School of Medicine, 580 Mikazura, Wakayama 641-0011, Japan; 5 Department of Ophthalmology, Yamaguchi University Graduate School of Medicine, Minami-Kogushi 1-1-1, Ube, Yamaguchi 755-8505, Japan

**Keywords:** CRP2, 3D structure, myocardin-related transcription factor, myofibroblast, cancer-associated fibroblasts

## Abstract

Inflammatory response induces phenotypic modulation of fibroblasts into myofibroblasts. Although transforming growth factor-βs (TGF-βs) evoke such transition, the details of the mechanism are still unknown. Here, we report that a LIM domain protein, cysteine-and glycine-rich protein 2 (CSRP2 [CRP2]) plays a vital role in the functional expression profile in myofibroblasts and cancer-associated fibroblasts (CAFs). Knock-down of CRP2 severely inhibits the expression of smooth muscle cell (SMC) genes, cell motility, and CAF-mediated collective invasion of epidermoid carcinoma. We elucidate the following molecular bases: CRP2 directly binds to myocardin-related transcription factors (MRTF-A/B [MRTFs]) and serum response factor (SRF) and stabilizes the MRTF/SRF/CArG-box complex to activate SMC gene expression. Furthermore, a three-dimensional structural analysis of CRP2 identifies the amino acids required for the CRP2-MRTF-A interaction. Polar amino acids in the C-terminal half (serine-152, glutamate-154, serine-155, threonine-156, threonine-157, and threonine-159 in human CRP2) are responsible for direct binding to MRTF-A. On the other hand, hydrophobic amino acids outside the consensus sequence of the LIM domain (tryptophan-139, phenylalanine-144, leucine-153, and leucine-158 in human CRP2) play a role in stabilizing the unique structure of the LIM domain.

## Introduction

Fibroblasts produce extracellular matrix (ECM) and play a role in wound healing. Although ECM productivity is low in the quiescent state, this function is activated when stimulated with cytokines such as transforming growth factor-βs (TGF-βs). These fibroblasts are known as myofibroblasts. Several types of cells are a source of myofibroblasts. Phenotypic modulation from fibroblasts to myofibroblasts is well-known. In this case, fibroblasts nearby the injured tissues undergo fibroblast-to-myofibroblast transition in response to inflammation. Transdifferentiation from epithelial cells to myofibroblasts is known as an epithelial-mesenchymal transition (EMT) (Morita *et al.*, 2007). These modulations are transient; once wound healing is completed, the recruited myofibroblasts undergo apoptosis (Pardali *et al.*, 2017; Karagiannis *et al.*, 2012). However, chronic activation of myofibroblasts is the cause of fibrotic diseases (Jun and Lau, 2018; Saika *et al.*, 2008). In addition, circulating bone marrow-derived stem cells and endothelial cells can be the source of myofibroblasts. Ectopic expression of smooth muscle cell (SMC) genes occurs in myofibroblasts. SMC genes include genes encoding actin, cell adhesion molecules, and collagens. The expression of α-SMA is a hallmark of myofibroblasts. EMT requires cooperative actions of Rho signals and myocardin-related transcription factors (MRTFs) such as MRTF-A and MRTF-B, which are transcriptional cofactors for serum response factor (SRF) and Smad (Gasparics and Sebe, 2018; Morita *et al.*, 2007; Posern and Treisman, 2006). However, the molecular basis for this remains unclear.

The myofibroblast-like cells localized in the tumor microenvironment are known as cancer-associated fibroblasts (CAFs) (Karagiannis *et al.*, 2012; Räsänen and Vaheri, 2010). The differentiation of fibroblasts into CAFs is similar to the fibroblast-to-myofibroblast transition. In the case of a collective invasion of epithelial cancer, CAFs aggregate with the cancer cells at the invasion front, and the aggregates migrate into the adjacent tissues (Karagiannis *et al.*, 2012). Thus, CAFs serve as the leader cells of the invasion.

Although MRTFs are localized primarily in the cytoplasm, they translocate into the nucleus transiently as a response to Rho signaling. This translocation activates the SRF-dependent transcription, which is causative for EMT (Morita *et al.*, 2007). We have previously reported that MRTFs accumulate in the nucleus in arterial endothelial cells in vivo and cultured human aortic endothelial cells (HAoECs). However, the SRF-mediated transcription is suppressed (Hayashi *et al.*, 2015). In myofibroblasts transited from primary-cultured human skin fibroblasts (HSFs) and CAFs, MRTFs constitutively locate in the nucleus and activate the SRF-mediated transcription. These findings suggest a cell type-dependent regulation of MRTFs.

Here, we found a novel regulatory mechanism of MRTFs by cysteine-and glycine-rich protein 2 (CSRP2 [CRP2]), which is a member of the CSRP family LIM domain protein (Weiskirchen *et al.*, 1995). Each member, CRP1, CRP2, or CRP3, plays significant roles in cell differentiation, cytoskeletal remodeling, and transcription (Wu *et al.*, 2014; Najwer and Lilly, 2005; Weiskirchen and Günther, 2003; Henderson *et al.*, 1999). Our findings are as follows: CRP2 directly binds to MRTF and SRF and stabilizes the MRTF/SRF/CArG-box complex to induce SMC gene expression; a three-dimensional (3D) structural analysis of CRP2 provides a detailed molecular mechanism for the CRP2-MRTF interaction.

## Materials and Methods

### Reagents and antibodies

Latrunculin B (LatB) and Y27632 were purchased from Caymen Chemical. TGF-β2 was purchased from PeproTec. Antibodies used in this study are as follows: anti-CRP2 (CSRP2) [HPA045617], anti-MRTF-B (MKL2) [HPA001286], anti-α-smooth muscle actin (α-SMA) [A2547], anti-β-actin [A5441], and anti-α-tubulin [T9026] antibodies and anti-FLAG M2 gel (Sigma); anti-haemagglutinin (HA) affinity matrix and anti-HA (3F10) antibody (Roche Applied Science); anti-CRP1 (CSRP1) antibody (Gene Tex, GTX114344); anti-collagen type I antibody (LifeSpan Bioscience, LSL-LB-1102); anti-DYKDDDDK (anti-Flag) antibody (Trans Genic, KO602-M); anti-Pan actin [#4968], and anti-SRF [#5147] antibodies (Cell Signaling Technology); anti-glyceraldehyde-3-phosphate dehydrogenase (GAPDH) antibody (Ambion, AM4300); anti-MRTF-A [sc390324], anti-cofilin [sc376476], anti-phospho-cofilin [sc271921] antibodies (Santa Cruz Biotechnology). Phalloidin and secondary antibodies conjugated to Alexa Fluor 568 or Alexa Fluor 488 were purchased from Molecular Probes. For the chromatin immunoprecipitation (ChIP) assay, we used a polyclonal anti-MRTF-A antibody that we previously produced (Hayashi *et al.*, 2015).

### Plasmids

We already constructed the expression plasmids for Flag-tagged mouse MRTF-A, its derivatives, and Myc-tagged human SRF (Hayashi and Morita, 2013). The expression plasmid for MRTF-A indicates a MAL fl-expressing plasmid. We constructed the expression plasmid for Flag-MAL fl D477-C encoding C-terminal domain (477–1021 amino acids) by PCR-mediated mutagenesis. cDNA of human CRP2 (NM_001321.2) or human CSRP2 binding protein (CSRP2BP [CRP2BP]) (NM_020536.4) was amplified by RT-PCR and inserted into an expression plasmid, pCS2+ with the indicated tag. We similarly constructed the expression plasmids for the truncated HA-CRP2s [HA-CRP2ΔC (2–100 amino acids) and HA-CRP2ΔN (101–193 amino acids)] and mutant CRP2s and confirmed their sequences.

### Cell culture and transfection

Primary-cultured normal HSFs (CRL-2072) were purchased from ATCC. Primary-cultured HAoECs (C-12271) and HaCaT cells were purchased from PromoCell and CLS Cell Lines Service GmbH, respectively. Isolation and characterization of CAFs, which were approved by the appropriate institutional review board, were previously described (Tanaka *et al.*, 2015). HAoECs were cultured as described elsewhere (Hayashi *et al.*, 2015). HSFs, CAFs, NIH3T3, HaCaT, and A431 cells were cultured in Dulbecco’s modified Eagle’s medium (DMEM) supplemented with 10% fetal calf serum.

We used Lipofectamine 3000 (Invitrogen) or ViaFect (Promega) for plasmid transfection. siRNAs against human MRTF-A (Hs_MKL1_6467), MRTF-B (Hs_MKL2_6806), CRP2 (Hs_CSRP2_1147), CRP1 (Hs_CSRP1_3285), α-SMA (Hs_ACTA2_7016), and a scrambled siRNA (control [cntl] siRNA) for control experiments were purchased from Sigma. Different type siRNAs are as follows: MRTF-A (SASI_Hs01_00156468), MRTF-B (SASI_Hs02_00345053), CRP2 (SASI_Hs02_00331621), and CRP1 (SASI_Hs01_00133287). We used Lipofectamine RNAiMAX (Invitrogen) for siRNA transfection.

### RT-qPCR

Total RNAs isolated from cultured cells were converted to cDNA using PrimeScript 1st strand cDNA Synthesis Kit (Takara Bio). We performed RT-qPCR using the Brilliant III Ultra-Fast SYBR QPCR MasterMix (Agilent Technologies) and a LightCycler Nano (Roche Life Science). Each mRNA level was normalized to GAPDH mRNA. These analyses were performed in triplicate and repeated three times. The levels in control cells (cntl siRNA-transfected cells and/or vehicle-stimulated cells) were set at 100% (means ± SEMs of the results from at least three independent experiments). The specific primers are described in Supplemental Data.

### Immunocytochemistry

Fluorescent images of immunocytochemistry were collected using a Biorevo BZ-X700 fluorescence microscope (Keyence). Subcellular localization of MRTFs was categorized into three groups: nuclear-specific localization (N); diffuse distribution in the nucleus and the cytoplasm (NC); and cytoplasmic localization (C) (Hayashi *et al.*, 2015; Hayashi and Morita, 2013). These classifications depend on nuclear/cytoplasmic (N/C) signal intensity: N/C >75%, nuclear-specific localization; N/C = 50–75%, diffuse distribution in the nucleus and cytoplasm; N/C <50%, cytoplasmic localization. For each experiment (at least three independent experiments), 100–200 cells were analyzed. We determined the proportions of the respective localization patterns (means ± SEMs).

### Immunoblot (IB) analysis

Whole-cell lysates or protein samples were separated by SDS-PAGE and then transferred to poly vinylidene fluoride (PVDF) membranes (Immobilon-P) (Merck Millipore). The membranes were incubated with the indicated antibodies. Target proteins on the membranes were detected by a luminescent reagent, Immuno-Star Zeta or LD (FUJIFILM Wako Pure Chemical Corporation), and C-DiGit Blot Scanner (LI-COR Biosciences). In the IB analysis using whole-cell lysates, we used α-tubulin and GAPDH as loading controls. These analyses were performed at least three times, and IB signal intensities were compared and quantified with each control data as 100% (mean ± SEMs).

### Wound healing assay

Cells were grown to 70–80% confluency, transfected with the indicated siRNA, and cultured for another 2–3 days. In the case of TGF-β2 stimulation, HSFs were cultured in low serum (0.3% fetal calf serum) medium after siRNA transfection and then stimulated with TGF-β2 (2 ng/ml). Confluent cells were scratch-wounded with a 20 μl pipette tip. Cell migration was monitored using a low-light inverted Olympus microscope (CKX 41) coupled with a monitoring system, CellPad-E (Tucsen) at 0 and 10 hours after scratching. Consecutive 4–5 images obtained by the above system were analyzed using NIH ImageJ software to quantify the migration area. The details are as follows. We traced the area that was not cell-migrated (immediately after scratching and after 10 hours) with Freehand Line and measured it to calculate the cell migration area. Percentages indicate the relative migration areas normalized by the control cell migration (cntl siRNA-transfected cells), which was set at 100% (means ± SEMs of the results from five consecutive places). We repeated these assays two and three times.

### CAF-mediated collective cancer cell invasion assay

Matrigel plate coating was performed according to the manufacturer’s instructions with some modifications. Briefly, Matrigel (250 μl/well) was placed in a 24-well plate and solidified at 37°C for 1 hour. Detailed procedures are as follows: mixtures of equal numbers (2–3 × 10^4^ cells) of CAFs transfected with either cntl siRNA or anti-CRP2 siRNA and untreated A431 cells were seeded on Matrigel.

### Promoter assay

Cells were transfected with a 3xCArG-Luciferase reporter plasmid (Morita *et al.*, 2007) (500 ng), pSV-β-gal (300 ng), and the indicated expression plasmids (100 ng) and then cultured for 48 hours. The cell extracts were subjected to luciferase and β-galactosidase assays using a luciferase assay kit (Promega) and β-galactosidase assay kit (Clonthec), respectively. Relative promoter activity was expressed in luminescence units normalized to the β-galactosidase activity of pSVβ-gal. The activities in control cells (the indicated plasmid-transfected cells) were set at 100% (means ± SEMs). We performed these assays in triplicate and repeated them at least twice.

### ChIP assay

ChIP assays were performed using ChIP assay kits (Merck Millipore) as described elsewhere (Hayashi *et al.*, 2015). Cross-linked chromatin was isolated from the indicated cultured cells transfected with either control siRNA or anti-CRP2 siRNA. DNAs isolated from input chromatin fragments and precipitated chromatin fragments by a control antibody or anti-MRTF-A antibody were subjected to qPCR using the indicated primers flanking the SRF-binding sites in the human α-SMA gene (Fig. S5C).

### Protein-protein interaction analysis

We performed in vitro protein-protein interactions according to previously described methods (Hayashi *et al.*, 2015; Hayashi *et al.*, 2014). The indicated proteins were synthesized using a TNT SP6 high-yield expression system based on an optimized wheat germ extract (Promega). The immunoprecipitation (IP) mixtures (500 μl) containing the indicated in vitro translated proteins were subjected to IP/IB analysis. For these analyses, 3.3% of the input proteins and 22.2% of the IP proteins were subjected to IB, respectively. We similarly performed IP/IB analysis with the whole-cell extracts. We repeated these analyses three times and quantified the IP/IB signal intensities using NIH ImageJ software. Relative binding affinity was calculated as follows: the ratio of the signal intensity of the Co-IP protein signal to the IP protein signal was normalized (mean ± SEMs) by the value of the control experiment, which was 100%.

### DNA affinity binding assay

A biotinylated probe for 3xCArG containing three SRF-binding sites (Morita *et al.*, 2007) was incubated with Streptavidin M-280 Dynabeads (Invitrogen) according to the manufacturer’s instructions as described previously (Hayashi *et al.*, 2015). In brief, the resulting Dynabeads-3xCArG and the indicated in vitro translated proteins were incubated at 4°C for 3 hours with rotation. Proteins bound to the probe were detected by IB analysis. We used NIH ImageJ software to quantify the signal intensity of each pull-down/IB. Binding affinity in the control experiments was set to 100% (mean ± SEM of results from at least three independent experiments).

### Searching for a protein-binding cavity in CRP2 by 3D structure analysis

Searching for the possible protein-binding cavity was performed by the program MAKE_RECEPTOR (Ver. 3.2.0.2) of OEDocking Suite (3.2.0.2; Openeye Scientific software). The original 3D structure of quail CRP2 was retrieved from the Protein Data Bank (PDB), NMR structure of the C-terminal LIM domain in solution from quail cysteine-rich protein CRP2 (PDB ID 1qli) [82–194 amino acids] (Konrat *et al.*, 1997). The sequence alignments of human and quail CRP2 proteins are shown in Fig. 7A. The sequences of the carboxyl-terminal LIM domain of quail CRP2 and the corresponding sequence in human CRP2 are underlined.

### Molecular dynamics (MD) simulation

Details are described in Supplemental Data.

### Statistical analysis

Results are given as means and standard errors. We statistically analyzed the data using a two-tailed paired student t-test and used an ANOVA with Bonferroni correction for the multi-data. The significance level was set at 0.05.

## Results

### Suppression of the myofibroblast phenotype of HSFs by decreased CRP2 expression

**Fig. 1:**
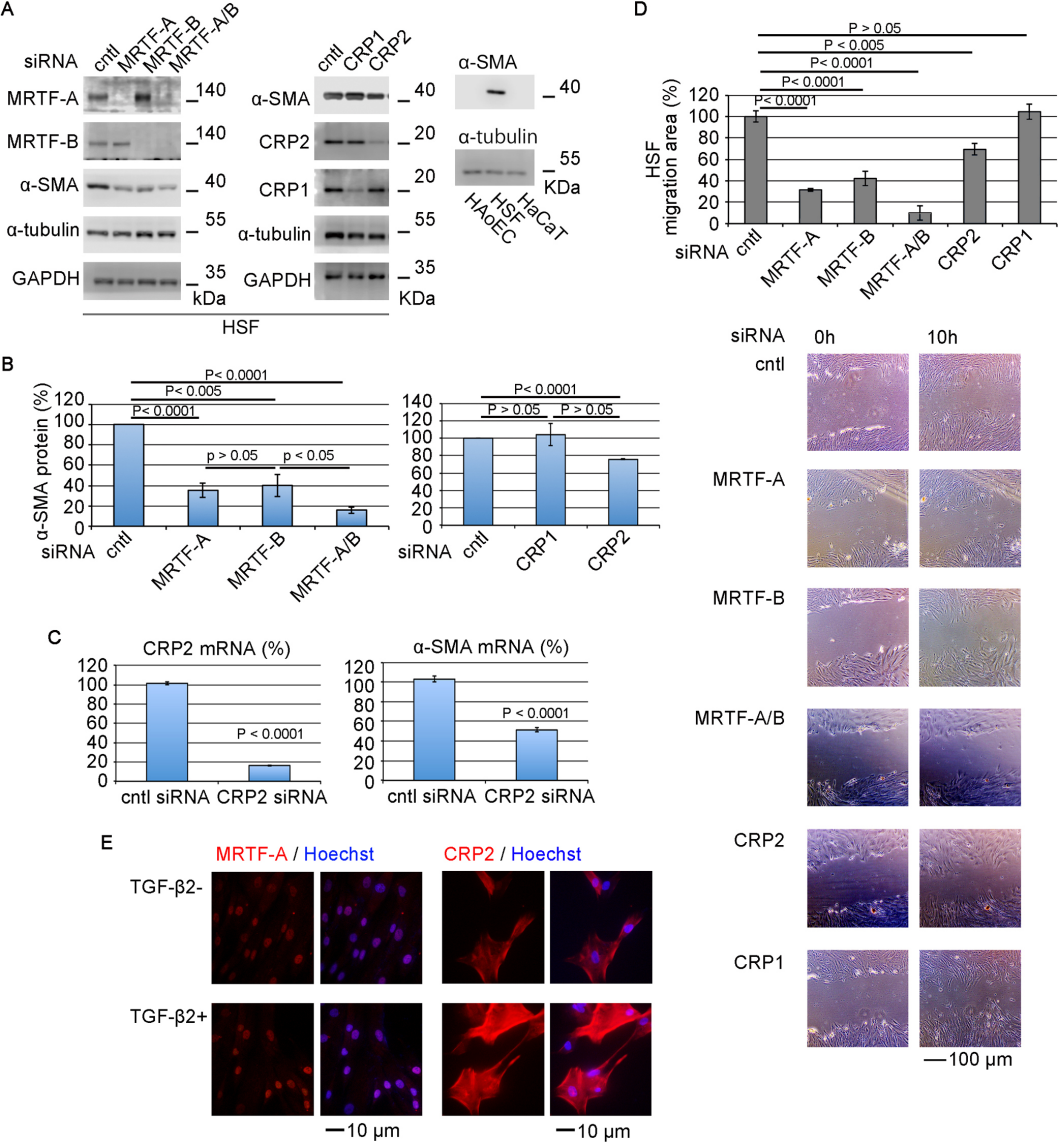
Effect of MRTF on the myofibroblastic phenotype of HSFs (A–D) HSFs were transfected with the indicated siRNAs and then cultured until confluent. IB analysis with whole-cell lysates (A), quantification of α-SMA protein expression (B), RT-qPCR analysis of CRP2 and α-SMA mRNAs expression (C), and wound healing assay (D). The expression levels of α-SMA protein and CRP2 and α-SMA mRNAs in HSFs transfected with control (cntl) siRNA were set at 100% (means ± SEMs of three independent experiments) (B, C). ANOVA shows a significant difference in the expression of α-SMA protein (p<0.0001 for the left and p = 0.0153 for the right, respectively). The migration area of control cells (cntl siRNA-transfected cells) was set at 100% (means ± SEMs of the results from five consecutive places). Representative images of wound healing assays are shown under the graph (D). ANOVA shows a significant difference in the wound healing assay (p<0.0001). (E) Subcellular localization of CRP2 and MRTF-A in HSFs in the absence or presence of TGF-β2-stimulation. HSFs were cultured in the presence of vehicle (PBS containing 0.1% BSA) or TGF-β2 (2 ng/ml) for 1 day. Cells were stained with anti-MRTF-A (red) and anti-CRP2 (red) antibodies, as well as Hoechst 33258 (blue). These are representative images from at least three independent experiments.

In vivo, fibroblasts reside in the stroma surrounded by the ECM, and their adhesion to the ECM plays a significant role in maintaining the quiescent phenotype. However, in vitro culture (on culture dishes), primary cultured HSFs exhibit a myofibroblastic phenotype (α-SMA-positive), but HAoCs or HaCaT cells are α-SMA-negative (Fig. 1A). The expression of SMC genes such as α-SMA depends on the SRF/CArG-box-mediated transcriptional regulation, which requires several cofactors for SRF (Gasparics and Sebe, 2018; Posern and Treisman, 2006; Morita *et al.*, 2007). They include MRTFs, CRP1, and CRP2 (Wu *et al.*, 2014; Chang *et al.*, 2003; Henderson *et al.*, 1999). We focused on these transcription factors to investigate the myofibroblastic phenotype of HSFs. As shown in Fig. 1A, B, and D, knock-down (KD) of either or both MRTF-A and MRTF-B with small interfering RNA (siRNA) reduces α-SMA expression and motility. The KD of CRP2 but not CRP1 also reduces the expression of α-SMA and motility. Different type of siRNAs causes similar results (Fig. S1). These findings suggest that CRP2 is involved in the phenotypic transition from HSFs to myofibroblasts. In HSFs with or without TGF-β2, MRTF-A mainly accumulates in the nucleus, and CRP2, on the other hand, is widely distributed in the cytoplasm and nucleus (Fig. 1E).

As MRTF-A constitutively locates in the nucleus in HSFs, we further analyzed the subcellular localization of MRTFs (Fig. S2). To address the significance of actin dynamics in the nuclear accumulation of MRTF-A, we examined the effect of either the actin polymerization inhibitor latrunculin B (LatB) or the Rho-kinase inhibitor Y27632. Treatment with LatB significantly decreases F-actin fibers, but only slightly affects the nuclear localization of MRTF-A. Treatment with Y27632 causes similar results (Fig. S2A, B). Phosphorylation of cofilin (Fig. S2C) and F-actin intensities become weak, but the nuclear localization of MRTF-A is not affected. MRTF-B diffusely locates throughout the cytoplasm and nucleus in the absence or presence of either LatB or Y27632 (Fig. S2A, B). KD of CRP2 does not affect the subcellular localization of MRTFs in HSFs (Fig. S2D, E). Therefore, the nuclear accumulation of MRTF does not depend on actin dynamics in HSFs. In our previous study (Hayashi and Morita, 2013), we reveal the differences in the nuclear export mechanism between MRTF-A and myocardin; constitutive nuclear localization of myocardin is due to its higher binding affinity to the importin α/β1 heterodimer and lower binding affinity to CRM1. In HSFs, the binding of some proteins to the nuclear export signal of MRTF-A may inhibit the CRM1-binding.

### Role of CRP2 in cellular function of TGF-β-stimulated HSFs

**Fig. 2:**
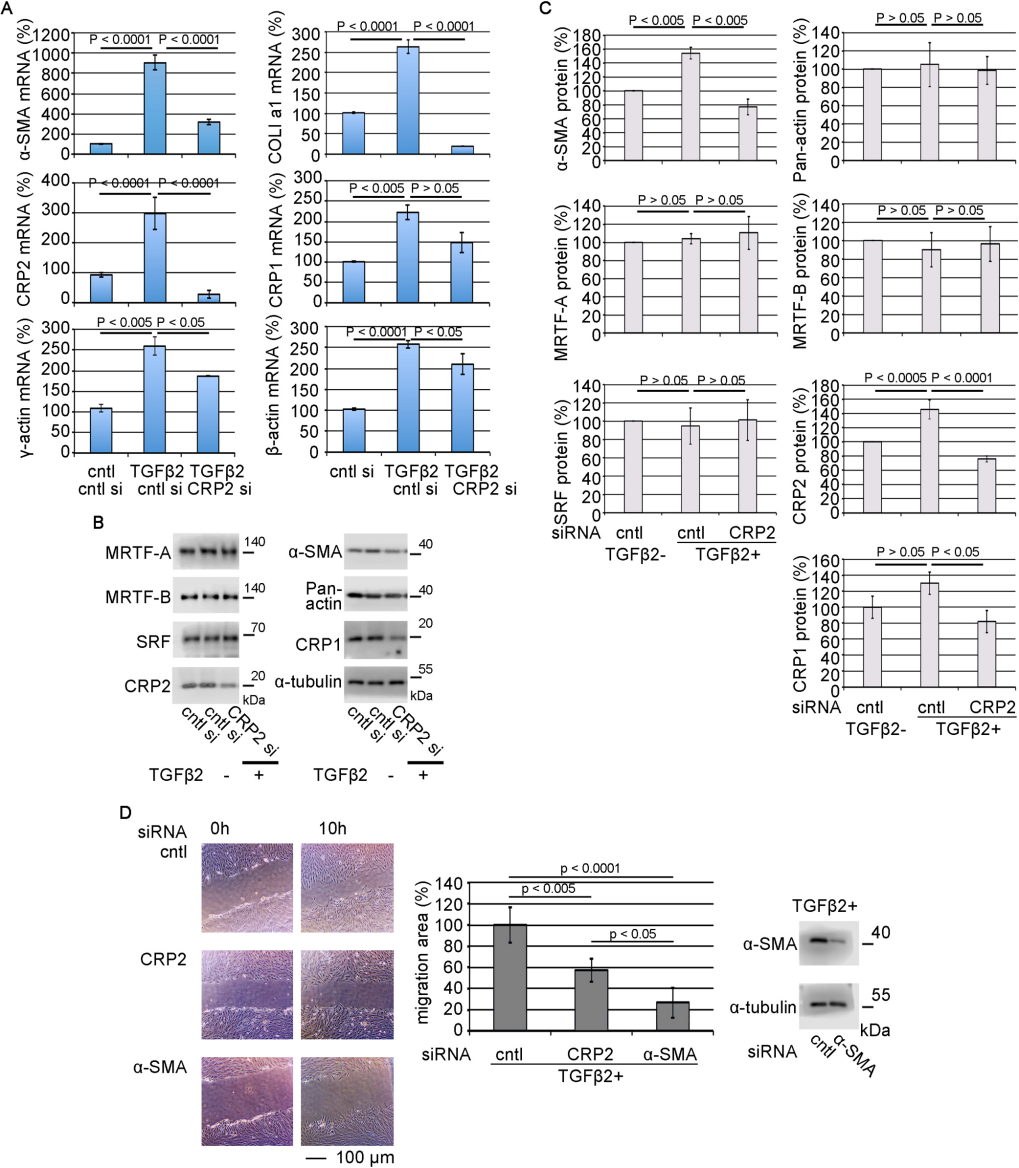
Roles of CRP2 in the myofibroblastic phenotype of TGF-β2-stimulated HSFs HSFs were transfected with the indicated siRNA and then cultured for 2 days. For the last 24 hours, HSFs were cultured with either vehicle (PBS containing 0.1% BSA) (control [cntl]) or 2 ng/ml TGF-β2. (A) RT-qPCR quantified the expression levels of the indicated actin isoforms, COLI a1, CRP1, and CRP2 at mRNA levels. Their expression in HSFs transfected with control siRNA (cntl si) in the absence of TGF-β2 was set at 100% (mean ± SEM of three independent experiments). ANOVA shows a significant difference in each RT-qPCR analysis (p<0.005). (B) IB analysis with whole-cell lysates from HSFs cultured under the indicated conditions as described above. (C) The graph shows the quantification of α-SMA, total actin (pan-actin), MRTF-A, MRTF-B, SRF, CRP2, and CRP1 proteins. Their expression levels in HSFs transfected with control siRNA in the absence of TGF-β2 were set at 100% (means ± SEMs of three independent experiments). ANOVA shows a significant difference in the expression of each of α-SMA, CRP2, and CRP1 proteins (p<0.01) but not in others (p>0.05). (D) Roles of CRP2 and α-SMA in HSF motility. HSFs were transfected with each of the indicated siRNAs and cultured TGF-β2-stimulated conditions. Confluent cultures were subjected to wound healing assay. Representative images of wound healing assays are shown (left panel). The middle graph shows the quantification of this assay. The migration area of control cells (cntl siRNA-transfected cells) was set at 100% (means ± SEMs of the results from five consecutive places). ANOVA shows a significant difference in the wound healing assay (p<0.01). IB analysis for monitoring the downregulation of α-SMA protein (right panel).

Since CRP2 is involved in the phenotypic transition from HSF to myofibroblasts, we examined how CRP2 is involved in the cellular function of TGF-β-stimulated HSFs. TGF-β2 activates the expression of SMC genes, CRP1, CRP2, TGF-β1, and TGF-β2 mRNAs, but not TGF-β3 mRNA. KD of CRP2 significantly reduces the TGF-β2-induced upregulation of α-SMA, COLI a1, CRP2, CRP1, β-actin, γ-actin, TGF-β2, and TGF-β1 mRNAs (Fig. 2A and Fig. S3). The expression profile of α-SMA protein is similar, but the total actin protein (pan-actin) expression is not suppressed (Fig. 2B, C). TGF-β2-stimulation and KD of CRP2 have little effect on the expression levels of MRTF and SRF (Fig. 2B, C). We then examined the KD effect of CRP2 or α-SMA on the motility of TGF-β2-stimulated HSFs. KD of CRP2 or α-SMA inhibits HSF migration (Fig. 2D). In particular, the KD effect of α-SMA is more inhibitory than that of CRP2, suggesting that up-regulation of α-SMA by TGF-β2 is involved in cell motility of myofibroblasts transited from HSFs. Therefore, CRP2 plays a critical role in maintaining the myofibroblastic features of TGF-β2-stimulated HSFs.

Since GATA family transcription factors are involved in the SRF-mediated transcription in vascular smooth muscle cells (VSMCs) (Chang *et al.*, 2003; Nishida *et al.*, 2002), we examined their expression profiles in HSFs. Although TGF-β2-stimulation and KD of CRP2 do not affect the expression of GATA5 and GATA6, TGF-β2-stimulation inhibits GATA4 expression (Fig. S3). The SRF/CRP2/GATA-mediated transcription in VSMCs requires CRP2BP, which acts as a histone acetyltransferase (Ma *et al.*, 2017). As with GATA5 and GATA6 expression, the effect of TGF-β2-stimulation on CRP2BP expression is less pronounced. However, KD of CRP2 slightly but significantly decreases its expression (Fig. S3). Thus, the expression profile of the GATA family does not correlate with that of the CRP2 and SMC genes.

### CRP2-dependent CAF functions

**Fig. 3:**
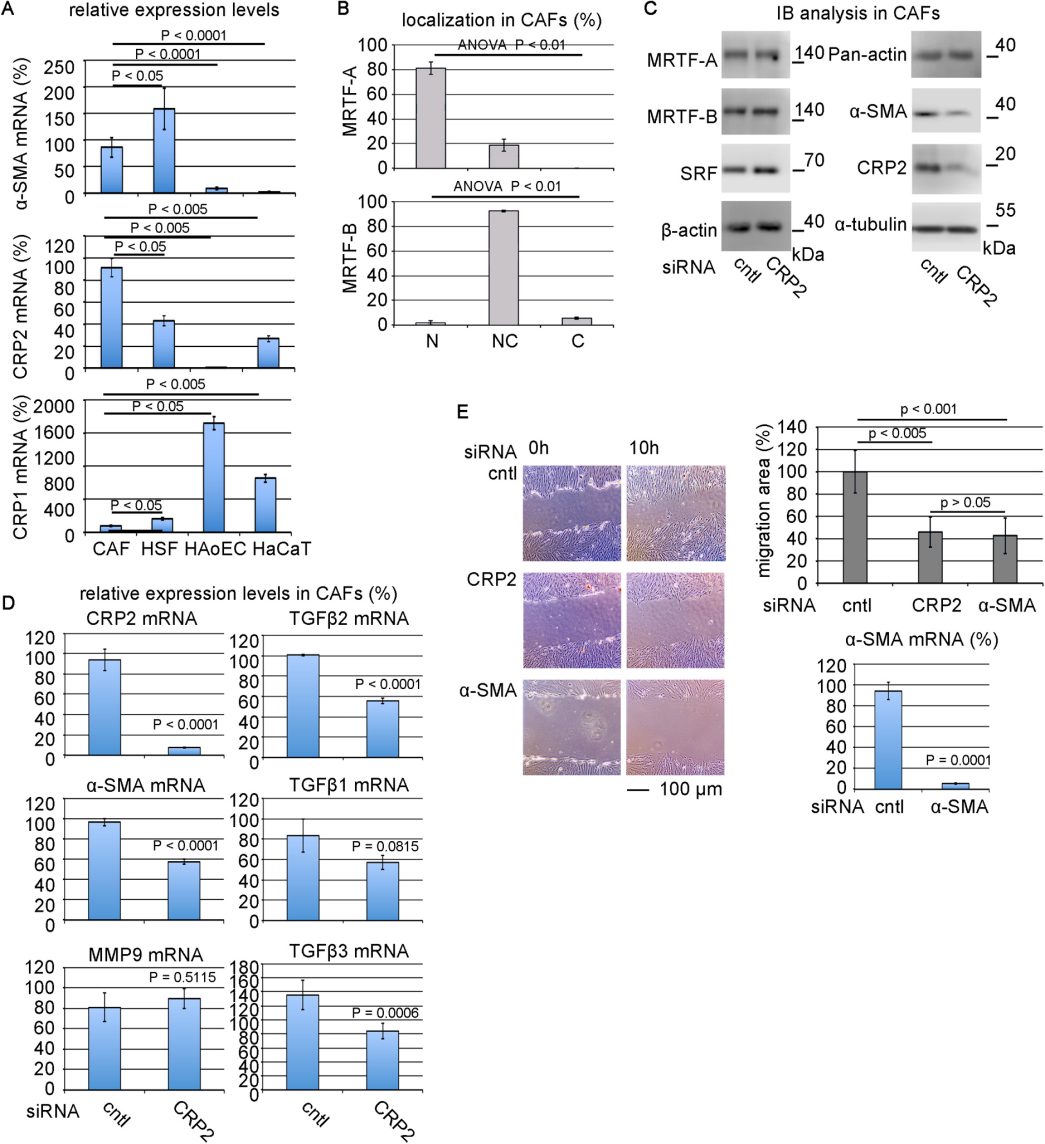
Role of CRP2 in the function of CAFs (A) Comparison of the expression levels of α-SMA, CRP1, and CRP2 among the indicated cells by RT-qPCR. Their expression levels in CAFs were set at 100% (means ± SEMs of three independent experiments). ANOVA shows a significant difference in each RT-qPCR analysis (p<0.0001). (B) Subcellular localization of MRTF-A and MRTF-B in CAFs. Cells were stained with either anti-MRTF-A antibody or anti-MRTF-B antibody and Hoechst 33258. Images were analyzed as described in Materials and Methods. Subcellular localization of MRTFs is categorized into three groups: nuclear-specific localization (N); diffuse distribution in the nucleus and the cytoplasm (NC); and cytoplasmic localization (C). ANOVA shows significant differences in the subcellular localization of MRTF-A and MRTF-B. Multiple comparisons of the subcellular localization of MRTF-A and MRTF-B are as follows: pair N-C, p<0.0001; pair N-NC, p = 0.0007; pair NC-C, p = 0.0179 for MRTF-A; pair N-C, p = 0.1872; pair N-NC, p<0.0001; pair NC-C, p<0.0001 for MRTF-B. (C, D) Effect of the KD of CRP2 on the expression of SMC genes and related genes in CAFs. CAFs transfected with each of the indicated siRNAs were cultured for 2 days. IB (C) and RT-qPCR (D) analyses. The respective mRNA expression levels in CAFs transfected with cntl siRNA were set at 100% (means ± SEMs of three independent experiments). (E) Roles of CRP2 and α-SMA in CAF motility. CAFs were transfected with each of the indicated siRNAs. Confluent cultures were subjected to wound healing assay. Representative images of wound healing assays are shown (left panel). The right upper graph shows the quantification of this assay. The migration area of control cells (cntl siRNA-transfected cells) was set at 100% (means ± SEMs of the results from five consecutive places). ANOVA shows a significant difference in the wound healing assay (p = 0.0106). RT-qPCR analysis for monitoring the downregulation of α-SMA mRNA (right lower panel).

To further address the significance of CRP2 in the myofibroblastic features, we compared the expression levels of α-SMA, CRP1, and CRP2 in four different types of cells: CAFs, HSFs, HAoECs, and human keratinocytes (HaCaT cells). CAFs and HSFs markedly express α-SMA but not in HAoECs and HaCaT cells. CRP1 expression levels in CAFs and HSFs are low, while CAFs, HSFs, and HaCaT cells remarkably express CRP2 (Fig. 3A). Although the expression levels of GATA family members are low in CAFs and HSFs, the levels of GATAs 4 and 5 are high in HAoECs (Fig. S4). Subcellular localization of MRTFs in CAFs was similar to that in HSFs (Fig. 3B). Our previous study showed that MRTFs are in the nucleus in HAoECs but not in HaCaT cells (Hayashi *et al.*, 2015). These results suggest that α-SMA expression is closely related to the nuclear localization of MRTFs and the expression level of CRP2. Thus, we characterized the roles of CRP2 in the functional profiles of CAFs. KD of CRP2 significantly reduces the expression of α-SMA at protein and mRNA levels (Fig. 3C, D) and inhibits cell motility (Fig. 3E). KD of α-SMA similarly reduces cell motility (Fig. 3E), suggesting that the migration of CAFs mainly depends on α-SMA. Thus, α-SMA plays a role in the cell motility of HSFs and CAFs. However, in HSFs, the KD of CRP2 modestly inhibits cell motility compared with the KD of α-SMA (Fig. 2D). This result may be due to the differences in the expression levels of α-SMA in HSFs and CAFs (Fig. 3A): higher levels in HSFs and lower levels in CAFs. TGF-β2-stimulation further increases the expression of α-SMA and CRP2 in HSFs (Fig. 2A). Therefore, the down-regulation of α-SMA by the KD of CRP2 would be weak in HSFs rather than CAFs, resulting in modest inhibition of HSF motility. KD of CRP2 also decreased the expressions of TGF-β1, -β2, and -β3, but not matrix metalloproteinase 9 (MMP-9) (Fig. 3D).

**Fig. 4:**
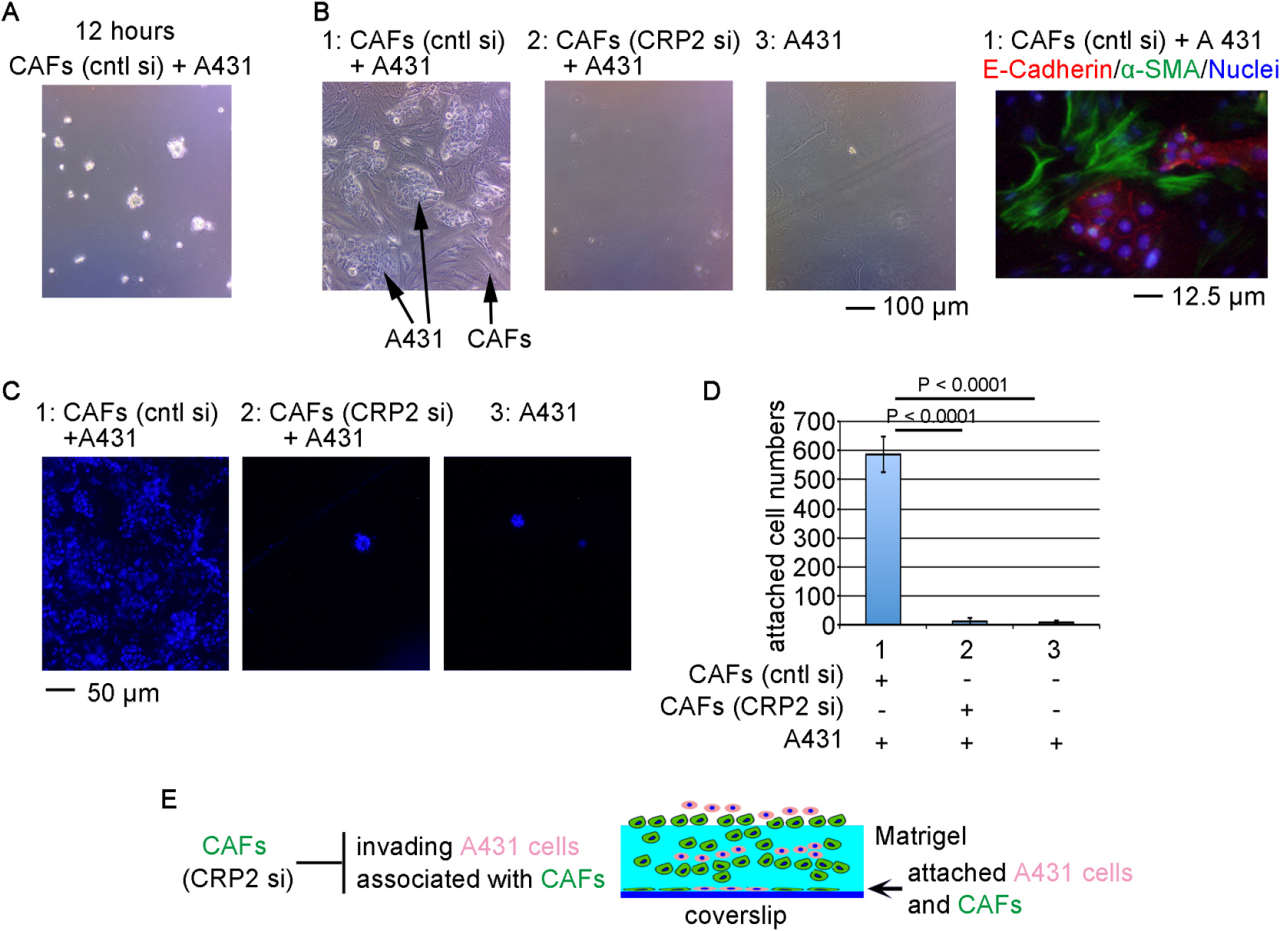
Inhibition of collective cancer cell invasion by depletion of CRP2 Role of CRP2 in the CAF-mediated collective invasion of A431 cells. CAFs were transfected with either control (cntl) siRNA or anti-CRP2 siRNA and then cultured for 4 days. siRNA transfection was repeated at 2 days after the first siRNA transfection. CAFs were collected and mixed with A431 cells. These cell mixtures and A431 cells were seeded on Matrigel and then co-cultured for 4 days. After fixation with 4% paraformaldehyde, the cultures were incubated in ice-cold x1 PBS (–) for a few hours to wash out Matrigel and then stained with the indicated antibodies and/or Hoechst 33258. For quantification of the collective invasion, the numbers of cell nuclei on the dish bottom were counted (means ± SEMs). These assays were performed in triplicate and repeated three times. (A) Phase contrast image of cells cultured for 12 hours. The image is focused on the surface of the Matrigel. (B) Phase contrast images of the respective 4-day cultures (left panels 1–3). These images were focused on the dish bottom (see Materials and Methods). Arrows indicate the colonies of cobblestone-like cells (A431 cells) and spindle-like cells (CAFs) on the dish bottom, respectively. The cultures were stained with anti-E-cadherin antibody (red), anti-α-SMA antibody (green), and Hoechst 33258 (blue) (right panel 1). (C, D) Quantification of the cells invaded through Matrigel and spread on the dish bottom. The cultures were stained with Hoechst 33258 (blue) (C), and the numbers of cell nuclei on the dish bottom were graphically represented (D). Each value represents the means ± SEMs of results from three independent experiments. ANOVA shows a significant difference in the invasion assay (p<0.0001). The schematic diagram shows the summary of this experiment (E).

We then addressed the role of CRP2 in the CAF-mediated collective invasion of a human epidermoid carcinoma cell line, A431 cells (Fig. 4). First, we introduced siRNA (control siRNA or siRNA against CRP2) into CAFs. A431 cells (untreated) and CAFs transfected with the respective siRNAs were co-cultured on Matrigel. These cells aggregate on the surface of Matrigel in 12 hours of cultures (Fig. 4A). In the control cultures on day 4, such cell aggregates arrive at the bottom of culture wells and form colonies, which consist of E-cadherin-positive cobblestone-like cells (A431 cells) and α-SMA-positive spindle-like cells (CAFs) (Fig. 4B, 1). In contrast, the cultures of CRP2-KD CAFs plus A431 cells or A431 cells alone hardly form such colonies (Fig. 4B, 2 and 3). The cells on the dish bottoms were stained with Hoechst 33258, and the blue puncta were quantified (Fig. 4C, D). These results suggest that the KD of CRP2 suppresses CAF-mediated collective invasion (Fig. 4E). Thus, CRP2 plays a significant role in this CAF function.

### Involvement of CRP2 in the transcriptional regulation by MRTF/SRF

**Fig. 5:**
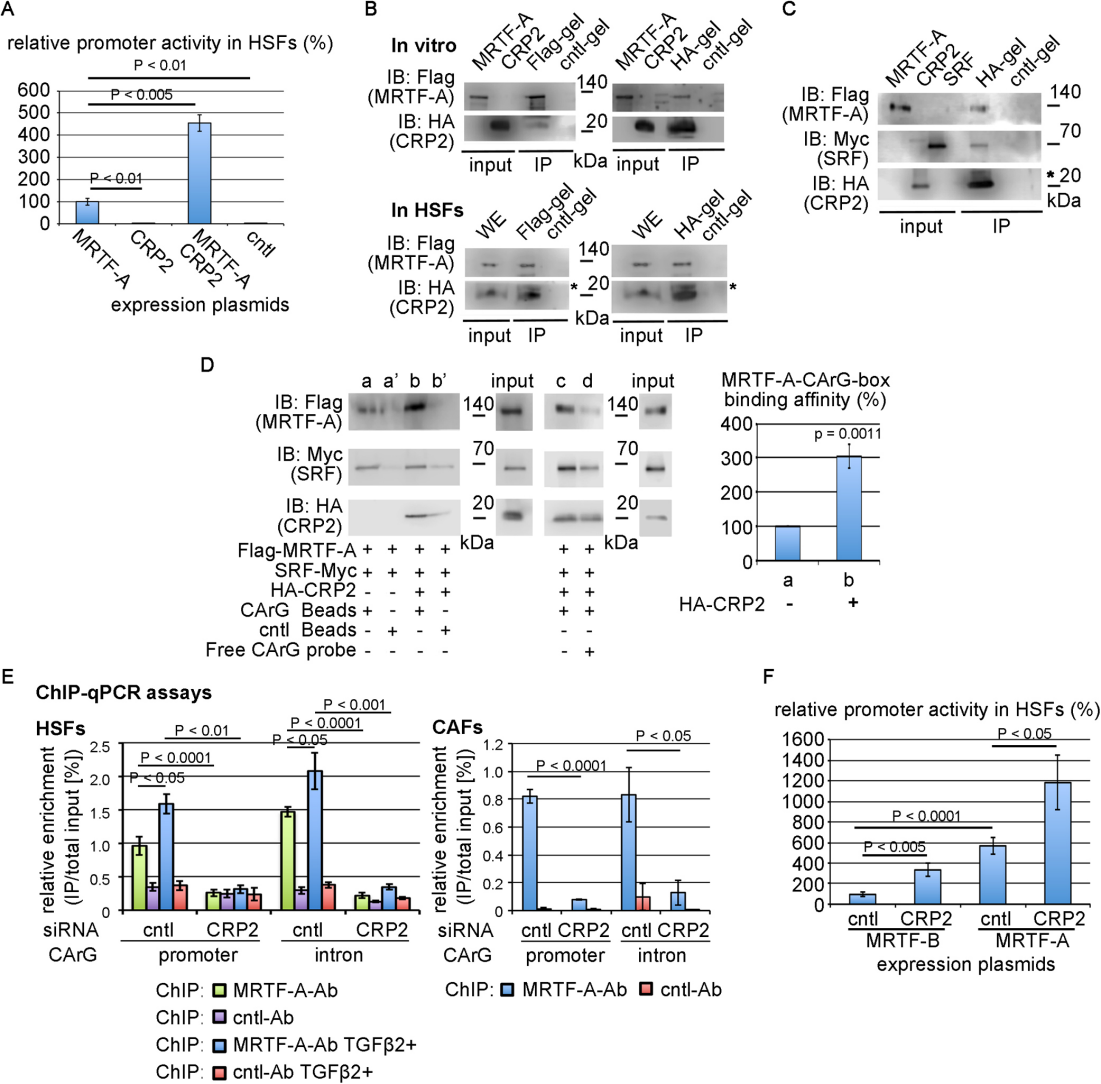
CRP2 as a cofactor for MRTF/SRF-mediated transcription (A) Promoter assay in HSFs. Cells were transfected with 3xCArG-Luciferase report plasmid, pSVβ-gal, and the indicated expression plasmids: Flag-tagged MRTF-A expression plasmid (MRTF-A), HA-tagged CRP2 expression plasmid (CRP2), and control plasmid (cntl). The luciferase activity induced by exogenous MRTF-A alone was set at 100%. ANOVA shows a significant difference in the promoter assay (p<0.0001). (B) Interaction between MRTF-A and CRP2. Mixtures of in vitro translated Flag-MRTF-A and HA-CRP2 proteins were immunoprecipitated with a control gel (cntl-gel), anti-HA-affinity matrix (HA-gel), or anti-Flag M2 affinity gel (Flag-gel) (upper panel). Whole-cell extracts (WE) from HSFs transfected with the expression plasmids for Flag-MRTF-A and HA-CRP2 were immunoprecipitated as described above (lower panel). IB indicates the proteins in each immuneprecipitate. (C) Interaction among MRTF-A, SRF, and CRP2. Mixtures of the indicated tagged proteins were immunoprecipitated and analyzed as described above. (D) DNA affinity binding assays using in vitro translated proteins. Mixtures of the indicated tagged proteins in the absence or presence of free 3xCArG were pulled down by either 3xCArG-Dynabeads (CArG-Beads) (a to d) or control Dynabeads (cntl Beads) (a' and b'). IB indicates the probe-bound proteins (left panel). The binding affinities of MRTF-A to 3xCArG-Dynabeads between reactions a and b were quantified (right panel). The binding affinity in reaction a was set at 100%. (E) ChIP-qRCR assays in HSFs and CAFs. Cross-linked chromatin was isolated from the indicated cultured cells transfected with either control siRNA or anti-CRP2 siRNA. In the case of HSFs, the siRNA transfected cells were stimulated either vehicle (PBS containing 0.1% BSA) or TGF-β2 (2 ng/ml) for 1 day. ChIP assays were performed with a control antibody (cntll-Ab) or anti-MRTF-A antibody (MRTF-A-Ab) as described in Materials and Methods. The graph shows the results of ChIiP-qRCR assays (means ± SEMs of three independent experiments). Fig. S5C shows the sense and antisense primers for the ChIP-qPCR. ANOVA shows a significant difference in each ChIP-qPCR assay (p<0.0001 for HSFs and CAFs). (F) Comparison of the synergistic effects between MRTF-A/CRP2 and MRTF-B/CRP2 on the CArG-box-mediated transcription. Promoter assay in HSFs was performed as described earlier. The luciferase activity induced by exogenous MRTF-B alone was set at 100%. ANOVA shows a significant difference in the promoter assay (p<0.0001). Asterisks (*) indicate the IgG light chain (B, C).

To address the significance of CRP2 in the transcription of SMC genes, we performed promoter assays in HSFs (Fig. 5A) and NIH3T3 cells with a feature of myofibroblasts (α-SMA-positive) (Fig. S5A). Potent CArG-box-dependent promoter activity is detected in cells expressing MRTF-A and CRP2 but not in cells expressing CRP2 alone, suggesting that CRP2 and MRTF-A synergistically activate the CArG-box-dependent transcription. CRP2 and MRTF-A similarly act in CAFs (Fig. S5B). We next examined the interaction among MRTF-A, CRP2, and SRF. First, we investigated the MRTF-A-CRP2 interaction by in vitro protein binding assay using in vitro translated tagged proteins or IP with cell extracts from HSFs expressing both tagged MRTF-A and CRP2. Immunoblot (IB) analysis reveals the direct interaction between MRTF-A and CRP2 (Fig. 5B). Furthermore, IP/IB analysis using three in vitro translated proteins (Flag-MRTF-A, SRF-Myc, and HA-CRP2) reveals that MRTF-A and SRF are in the immunoprecipitate by HA-antibody (Fig. 5C), suggesting a ternary complex of them. To confirm this, we performed a DNA affinity binding assay using the 3xCArG-box probe-coupled Dynabeads (3xCArG-box-Dynabeads). CRP2 does not alter the binding affinity of SRF to the 3xCArG-box probe but potently increases the binding of MRTF-A to the SRF/CArG-box probe complex (Fig. 5D). CRP2 does not directly bind to DNA (Chang *et al.*, 2003), but CRP2 associates with the 3xCArG-box probe mediated by its interaction with SRF and MRTF-A. A free 3xCArG-box probe addition suppresses such complex formation (Fig. 5D). These results indicate that CRP2 promotes the interaction between SRF and MRTF-A and stabilizes the complex consisting of MRTF-A/SRF/CArG-box. We then performed a chromatin IP (ChIP) assay using an anti-MRTF-A antibody to address whether endogenous interaction MRTF-A-CRP2 occurs in HSFs and CAFs. ChIP-PCR shows that KD of CRP2 significantly reduces the binding of MRTF-A to both CArG-boxes in the promoter and the first intron of the human α-SMA gene (Fig. 5E and Fig. S5C). TGF-β2-stimulation increases the binding of MRTF-A to both CArG-boxes in HSFs. This enhancement is due to the TGF-β2-induced up-regulation of CRP2 and supports the above results of the DNA affinity binding assay (Fig. 5D). CRP2 also activates the MRTF-B/SRF-mediated transcription, but the activation level is low compared with the MRTF-A/SRF-mediated transcription (Fig. 5F). In conclusion, CRP2 stabilizes the interaction between SRF and MRTF, leading to the activation of MRTF-A/SRF/CArG-box-mediated transcription.

### MRTF-A-CRP2 interaction

**Fig. 6:**
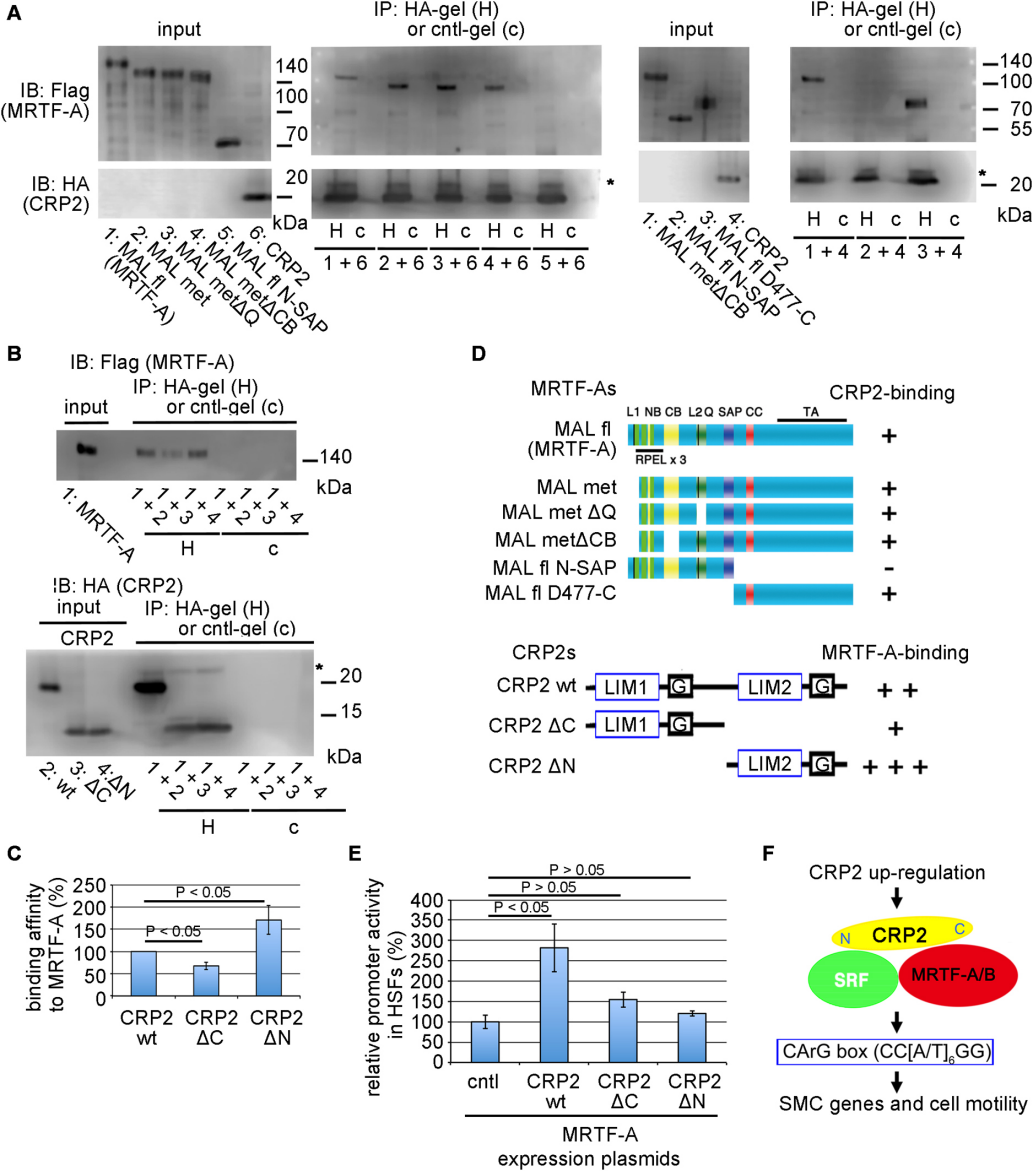
Interaction between MRTF-A and CRP2 (A–D) Identification of the CRP2-binding domain in MRTF-A (A) or MRTF-A-binding domain in CRP2 (B) by IP/IB analysis. Mixtures of the indicated tagged proteins (proteins numbered in the input panels) were immunoprecipitated with a control gel (cntl-gel [c]) or anti-HA-affinity matrix (HA-gel [H]). MAL fl stands for MRTF-A. Other MAL (MRTF-A) derivatives are described in a previously published study (Miralles *et al.* 2003). IB shows the respective MRTF-A derivative proteins coimmunoprecipitated with HA-CRP2 protein (A) or MRTF-A protein coimmunoprecipitated with either wild-type or mutant HA-CRP2 proteins (B). The binding affinities of the respective CRP2 proteins (wild-type [wt], ΔC mutant, and ΔN mutant) were quantified (C). The binding affinity of CRP2 wt was set at 100%. Asterisks (*) indicate the IgG light chain. ANOVA shows a significant difference in in the binding assay (p = 0.0009). (D) Schematic structures of wild-type and mutant MRTF-As and CRP2 and a summary of the binding assays. The schemas show the structures of MRTF-A derivatives and CRP2 derivatives. LIM1 and LIM2, LIM domain; G, glycine-rich domain. The relative binding affinities of these proteins are: –, no binding; + ~ +++, binding hierarchy. (E) Synergistic effects of MRTF-A and each of truncated CRP2s on the CArG-box-mediated transcription in HSFs. Cells were transfected with 3xCArG-Luciferase report plasmid, pSVβ-gal, and the indicated expression plasmids (see the legend of Fig. 5A). The luciferase activity induced by exogenous MRTF-A alone was set at 100%. ANOVA shows a significant difference in the promoter assay (p = 0.0009). (F) Model of CRP2-dependent transcriptional regulation in myofibroblasts.

To further examine the interaction between CRP2 and MRTF-A, we performed in vitro protein binding assay. Fig. 6A shows in vitro protein binding assays between each mutant MRTF-As and wild-type CRP2 proteins. As summarized in Fig. 6D, the C-terminal region of MRTF-A, containing a coiled-coil domain and a transactivation domain (TA) (477–1021 amino acids), is the CRP2-binding domain. CRP2ΔN exhibits potent binding affinity to MRTF-A compared to wild-type CRP2, but CRP2ΔC weakly binds to MRTF-A (Fig. 6B, C). Thus, the C-terminal half of CRP2 containing LIM2 (101–193 amino acids) is the main MRTF-A-binding domain. The binding affinity of CRP2ΔN to MRTF-A is higher than wild-type CRP2. This property may be due to shielding the MRTF-A-binding region in the C-terminal half of CRP2 by its N-terminal half. A previous study demonstrates that the N-terminal half of CRP2 containing LIM1 is the binding domain for SRF (Chang *et al.*, 2003). Taking these results together, we speculate that CRP2 may bind to both SRF and MRTF-A mediated by the N-terminal domain and the C-terminal domain, respectively. The promoter assay in HSFs strongly supports this view as the expression of each truncated CRP2 decreases the synergistic effect with MRTF-A (Fig. 6E). Fig. 6F shows a model of CRP2-dependent regulation in myofibroblasts.

The consensus sequences of the LIM domain are critical for the function of CRP2 (Chang *et al.*, 2003). The mutations into the consensus sequences of the LIM domain in LIM2 (Fig. S6A) reduce the binding affinity to MRTF-A and the MRTF-A/SRF-mediated transcription (Fig. S6B, C), suggesting that the consensus sequences of the LIM domain are critical for MRTF-A-CRP2 interaction.

### CRP2 function and its relationship to the 3D structure

**Fig. 7:**
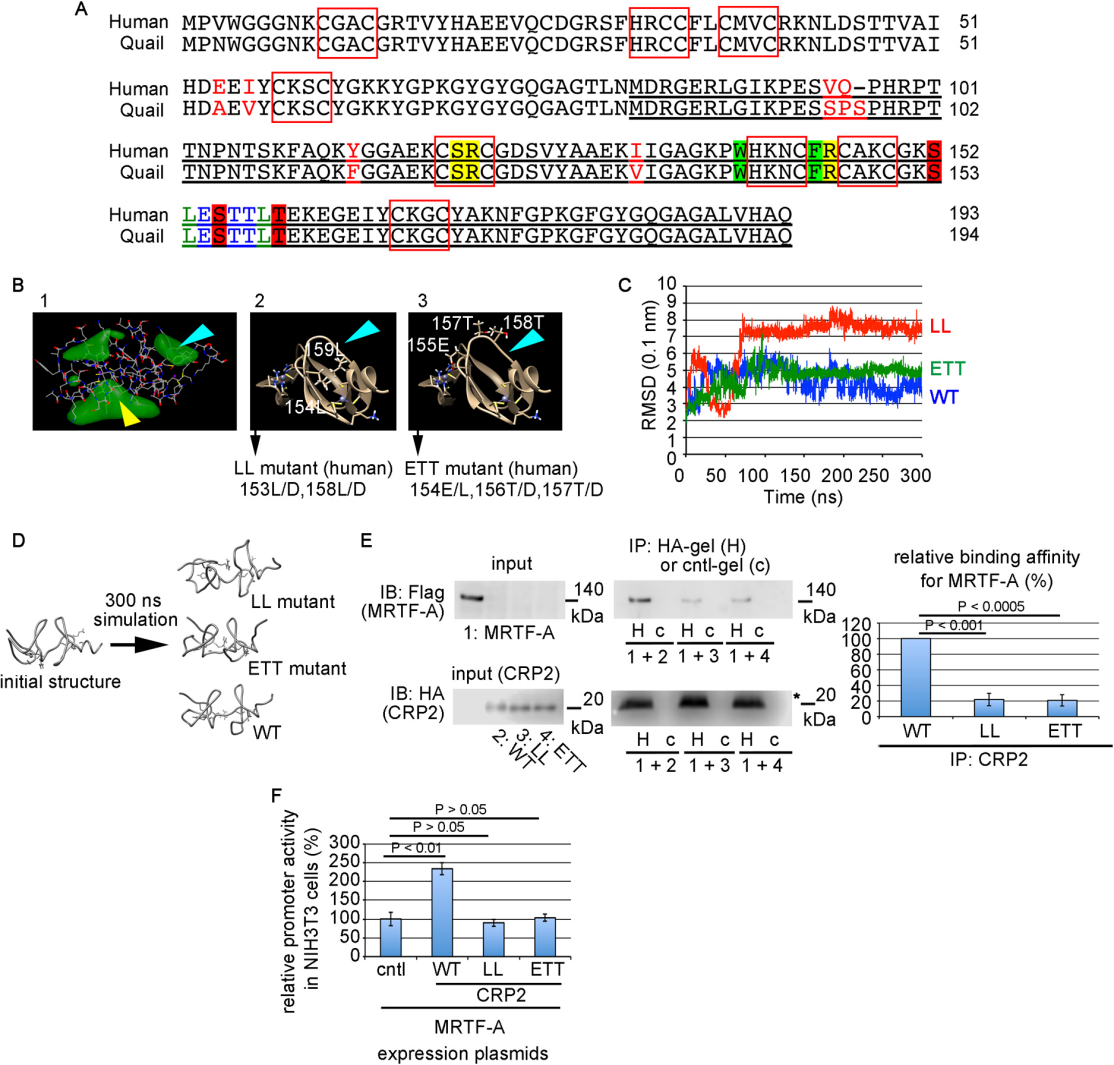
Identification of the MRTF-A-binding sites in the C-terminal half of CRP2 (A) Sequence alignments of human and quail CRP2 proteins. The red boxes indicate the consensus sequences of the LIM domain, and the red letters indicate human- or quail-specific amino acids. (B) The 3D structures of the C-terminal half of quail CRP2 (underlined sequence in A). Green cloudlike shapes indicate possible protein-binding cavities (panel 1). Yellow and light blue arrowheads indicate the largest and the second protein-binding cavities, respectively. Panels 2 and 3 show the 3D positions of the hydrophobic amino acids (154L, 159L in quail CRP2) and the polar amino acids (155E, 157T, 158T in quail CRP2) in the second protein-binding cavity, respectively (green letters and blue letters in A). (C, D) MD simulations of the C-terminal half of wild-type (WT) and the mutant CRP2 proteins (LL and ETT mutants). Graph (C) and schematic diagram (D) show their MD simulations. They are representative results from two independent simulations. (E, F) Examination of the biological functions of the LL mutant and the ETT mutant. The left panel and right graph in (E) show the interactions between MRTF-A and each of the CRP2 mutants using in vitro translated tagged proteins and the quantitative data, respectively. In vitro protein binding assays were performed as described in the legends of Figs. 5 and 6. In brief, mixtures of the indicated in vitro translated proteins (numbering proteins in the input panels) were subjected to IP/IB analysis. Percentages represent the binding affinity of each HA-CRP2 to Flag-MRTF-A normalized by the affinity of wild-type CRP2 (WT), which is 100% (mean ± SEMs of three independent experiments). Asterisk (*) indicates the IgG light chain. Synergistic effects of MRTF-A and each of the CRP2 mutants on SRF-mediated transcription (F). Promoter assay was performed in NIH3T3 cells. Cells were transfected with 3xCArG-Luciferase report plasmid, pSVβ-gal, and the indicated expression plasmids (see the legend of Fig. 5A). The luciferase activity induced by exogenous MRTF-A alone was set at 100%. ANOVA in each assay (E, F) shows a significant difference (p<0.0001).

Our findings suggest that CRP2 would be a novel drug discovery target for fibrotic diseases. We examined the relationship between the CRP2 function and its 3D structure to explore the above possibility. We characterized a 3D structure of the C-terminal half of quail CRP2 (82–194 amino acids) containing the MRTF-A-binding domain, as the 3D structure of quail CRP2 has been solved (Konrat *et al.*, 1997). Fig. 7A shows the alignments of human and quail CRP2 protein sequences. The sequences of the C-terminal half of quail and human CRP2 proteins are nearly identical (96%). Thus, the 3D structure of human CRP2 would be approximately equal to that of quail CRP2. To confirm this conjecture, we simulated the 3D structure of mutant quail CRP2, in which the quail-specific sequences are mutated to the human ones (mut [quail/human] = wild-type human CRP2). MD simulations show no significant structural difference between wild-type quail and human CRP2 proteins; both CRP2s exhibit a unique 3D structure that we named twin-peaks structure (Fig. S6D). Thus, we further promoted the MD simulation of quail CRP2 mutants corresponding to the human CRP2 mutants (mut1 and mut2 [Fig. S6A]). The introduction of mutations into the consensus sequences of the LIM domain destabilizes the twin-peaks structure (Fig. S6E), suggesting that the twin-peaks structure is critical for the function of CRP2.

The 3D structure of the C-terminal half of quail CRP2 predicts three possible protein-binding cavities indicated by the green cloudlike shapes (Fig. 7B). We first focused on the largest protein-binding cavity (Fig. 7B, panel-1, yellow arrowhead). Docking simulation suggests that the amino acids, 121S, 122R, and 146R in quail CRP2 (120S, 121R, and 145R in human CRP2; Fig. 7A, yellow-shaded letters) may play a role in a protein-protein interaction (Fig. S7A). The basis is the polarities of the amino acids composed of this protein-binding cavity. To assess this possibility, we analyzed the 3D structure of a mutant CRP2 protein, named SRR mutant, in which these polar amino acids were changed to hydrophobic amino acids (121S/A, 122R/V, 146R/A). MD simulations reveal no significant structural difference between wild-type quail CRP2 and the CRP2 SRR mutant (Fig. S7B, Video 1, and Video 2). We also prepared a mutant human CRP2 protein corresponding to the quail CRP2 SRR mutant and analyzed its biological functions. The binding affinity of the CRP2 SRR mutant to MRTF-A and the synergistic effect of this mutant and MRTF-A on SRF-mediated transcription are equivalent to wild-type CRP2 (Fig. S7C, D), indicating that these polar amino acids are unrelated to the functions of CRP2.

We next focused on the second protein-binding cavity (Fig. 7B, panel-1, light blue arrowhead). The amino acid sequence from 153S to 160T in quail CRP2 (152S to 159T in human CRP2) is within this protein-binding cavity (Fig. 7A). We introduced mutations into this sequence and analyzed the 3D structures of mutant CRP2 proteins. MD simulation suggests that two hydrophobic amino acids, 154L and 159L in quail CRP2 (153L and 158L in human CRP2) (Fig. 7A, green letters and Fig. 7B, panel-2), are critical for stabilizing the twin-peaks structure. A mutant quail CRP2, in which these hydrophobic amino acids are changed to aspartate (154L/D, 159L/D [LL mutant]), exhibits a quite different 3D structure (Fig. 7C, D, Video 1, and Video 3). In contrast, a significant difference in the 3D structure is not detected in a mutant quail CRP2, in which three polar amino acids (Fig. 7A, blue letters and Fig. 7B, panel-3) are changed to aspartate (155E/D, 157T/D, 158T/D [ETT mutant]) (Fig. 7C, D, Video 1, and Video 4). To evaluate the roles of these amino acids, we prepared two mutant human CRP2 proteins (LL mutant and ETT mutant) and examined their biological functions. These mutations reduce the binding affinity to MRTF-A and the synergistic effect on the transcription (Fig. 7E, F).

We also introduced another mutation into this second protein-binding cavity (153S, 156S, 160T in quail CRP2 [152S, 155S, 159T in human CRP2]) (Fig. 7A, red-shaded letters) or the flanking sequences of the LIM domain consensus sequences (140W and 145F in quail CRP2 [139W and 144F in human CRP2]) (Fig. 7A, green-shaded letters) and similarly analyzed. These mutations are as follows: the polar and hydrophobic amino acids were changed to aspartate (153S/D, 156S/D, 160T/D [SST mutant] and 140W/D, 145F/D [WF mutant]). MD simulation suggests that the SST mutation does not cause a significant structural change, but the WF mutation results in a drastic structural change (Fig. S7E, F, Video 1, Video 5, and Video 6). These mutations impaired the normal biological functions of CRP2 (Fig. S7G, H).

## Discussion

This study elucidated the critical roles of CRP2 in the functional expression of myofibroblasts and CAFs. The molecular mechanism is as follows: CRP2 binds to both SRF and MRTF and stabilizes the complex consisting of MRTF/SRF/CArG-box, leading to the activation of SMC gene expression followed by the promotion of cell motility. The 3D structural analyses revealed the MRTF-A-binding cavity in the C-terminal half of CRP2 (LIM2). This is the first report showing the relationship between the 3D structure of CRP2 and its binding to MRTF-A. Our findings suggest that CRP2 is a novel therapeutic target for fibrotic diseases.

### Functional regulation by CRP2 in myofibroblasts and CAFs

The neointimal formation is enhanced in the arterial balloon injury model in CRP2 knockout mice (Lilly *et al.*, 2010; Sagave *et al.*, 2008), indicating that CRP2 inhibits VSMC migration. In contrast, CRP2 plays a role in breast cancer cell invasion and metastasis (Hoffmann *et al.*, 2016). Similarly, CRP2 acts as a positive regulator for cell motility of myofibroblasts transited from HSFs and CAFs. These findings show cell-type-dependent differences in the functions of CRP2.

Distinct types of actins regulate cell motility in a cell type-dependent manner: γ-actin for rat lung fibroblasts (Dugina *et al.* 2009), β-actin for mouse embryonic fibroblasts (Bunnell *et al.*, 2011), and α-SMA for myofibroblasts from rat liver stellate cells (Rockey *et al.*, 2013). As α-SMA mainly controls the cell motility of HSFs and CAFs, the suppression of their motility by KD of CRP2 would be due to the depletion of α-SMA (Fig. 2D and Fig. 3E). Since the KD of CRP1 does not affect the expression of α-SMA, we speculate that the KD of CRP1 does not affect HSF motility (Fig. 1 and Fig. S1). We also demonstrate that CRP2 plays a critical role in CAF-mediated collective invasion of A431 cells (Fig. 4). Although MMP-9 regulates metastatic progression in cancer invasion (Daniele *et al.*, 2016), the KD of CRP2 hardly affects the expression of MMP-9 in CAFs (Fig. 3D). Therefore, the KD of CRP2-induced inhibition of such collective invasion is due to reduced CAF motility caused by the depletion of α-SMA.

These findings raise the question of whether CRP2 correlates to the onset of EMT. Our ongoing study suggests that CRP2 also plays a pivotal role in the onset of EMT (manuscript in preparation).

### Roles of CRP2 as a transcriptional cofactor

The regulatory model (Fig. 6F) represents the molecular basis of our conclusion: CRP2 directly binds to both SRF and MRTF and stabilizes the MRTF/SRF/CArG-box complex to promote the transcription of the SMC genes. In many cases, the transcriptional cofactors activate transcription mediated through the recruitment of epigenetic factors. However, CRP2-mediated transactivation does not require the recruitment of epigenetic factors. A transcription factor sometimes inhibits the transcription regulated by other transcription factors by forming a heterodimer or multimer. For example, Inhibitor of differentiation or Msx families associate with MyoD family or SRF/myocardin, leading to inhibition of the transcription mediated by E-box or CArG-box (Perk *et al.*, 2005; Hayashi *et al.*, 2006). In contrast, CRP2 acts as an activator. Although CRP2BP acts as an epigenetic factor (Ma *et al.*, 2017), our preliminary study suggests that CRP2BP also play a role as an adaptor protein that stabilizes the CRP2-mediated MRTF/SRF/CArG-box complex (manuscript in preparation). Transcriptional cofactors associated with epigenetic factors may also play a role in the adaptor proteins described above.

### Relationship between 3D structure and function of CRP2

The 3D structural analyses (Fig. 7, Figs. S6 and S7) reveal the so-called twin-peaks structure is critical for the CRP2-MRTF-A interaction. The hydrophobic amino acids (139W, 144F, 153L, and 158L in human CRP2), which locate outside the consensus sequences of the LIM domain, are essential for the formation of the second protein-binding cavity. We concluded that the amino acids (152S, 154E, 155S, 156T, 157T, and 159T in human CRP2), but not the above hydrophobic amino acids, play a vital role in direct interaction with MRTF-A. The rationale is as follows: 1) these amino acids are present in the second protein-binding cavity; 2) mutations in these amino acids (ETT and SST mutants) do not cause a drastic 3D structural change but severely impair the functions of CRP2 as a cofactor for the MRTF-A/SRF-mediated transcription. We infer that the polarity of these amino acids is significant for the interaction with MRTF-A. These data are applicable for drug discovery studies against fibrotic diseases.

## Data Availability

All data are contained within this manuscript.

## Author Contributions

K. Hayashi designed the study and performed the experiments with help from T. Morita and R. J. Labios. H. Miyata generated the CAFs used. S. Horoiwa, K. Mori, and Y. Nakagawa performed the 3D structure analyses. Y. Kobayashi, C. Yamashiro, F. Higashijima, T. Yoshimoto, and K. Kimura supervised this project. K. Hayashi wrote the manuscript. All authors reviewed and approved the final version of the manuscript.

## Competing Interest

The authors declare no competing financial interests.

## Abbreviations

α-SMA: α-smooth muscle actin
CAF: cancer-associated fibroblast
ChIP: chromatin immunoprecipitation
cntl: control
COLI: collagen type I
CRP: cysteine-and glycine-rich protein
DMEM: Dulbecco’s modified Eagle’s medium
ECM: extracellular matrix
EMT: epithelial-mesenchymal transition
GAPDH: glyceraldehyde-3-phosphate dehydrogenase
HA: haemagglutinin
HAoEC: human aortic endothelial cell
HSF: human skin fibroblast
IB: immunoblot
IP: immunoprecipitation
KD: knock-down
LatB: latrunculin B
MD: molecular dynamics
MMP-9: matrix metalloproteinase 9
MRTF: myocardin-related transcription factor
SMC: smooth muscle cell
SRF: serum response factor
3D: three-dimensional
TGF-β: transforming growth factor-β
VSMC: vascular smooth muscle cell

## Supplementary Material

Video 1. MD simulation of wild-type CRP2. MD simulation of the C-terminal half of wild-type quail CRP2 (82-194 amino acids). The movie corresponds to Fig. 7C, D, Fig. S6D, and Fig. S7B, E, F.

Video 2. MD simulation of CRP2 SRR mutant. MD simulation of the C-terminal half of mutant quail CRP2 [SRR mutant] (82-194 amino acids). The movie corresponds to Fig. S7B.

Video 3. MD simulation of CRP2 LL mutant. MD simulation of the C-terminal half of mutant quail CRP2 [LL mutant] (82-194 amino acids). The movie corresponds to Fig. 7C, D.

Video 4. MD simulation of CRP2 ETT mutant. MD simulation of the C-terminal half of mutant quail CRP2 [ETT mutant] (82-194 amino acids). The movie corresponds to Fig. 7C, D.

Video 5. MD simulation of CRP2 SST mutant. MD simulation of the C-terminal half of mutant quail CRP2 [SST mutant] (82-194 amino acids). The movie corresponds to Fig. S7E, F.

Video 6. MD simulation of CRP2 WF mutant. MD simulation of the C-terminal half of mutant quail CRP2 [WF mutant] (82-194 amino acids). The movie corresponds to Fig. S7E, F.
