# Supplementary material for: Role of CRP2-MRTF interaction in functions of myofibroblasts
Source: Cell Struct Funct. 2023 May 11;48(1):83–98. doi: 10.1247/csf.23004 (PMC10721955; doi:10.1247/csf.23004)
Supplement: Supplementary file 7 — Supplementary Materials [file csf_48_23004_7.zip › 48_23004_7.docx]

**Supplemental Data**

**Materials and Methods**

The specific primers for RT-qPCR

Molecular dynamics (MD) simulation

Supplemental Figures (S1-S7) and Videos (1-6)

Video 1. MD simulation of wild-type CRP2.

Video 2. MD simulation of CRP2 SRR mutant.

Video 3. MD simulation of CRP2 LL mutant.

Video 4. MD simulation of CRP2 ETT mutant.

Video 5. MD simulation of CRP2 SST mutant.

Video 6. MD simulation of CRP2 WF mutant.

**Materials and Methods**

**RT-qPCR**

The specific primers used in this study are as follows:

GAPDH sense primer, ACTCCTCCACCTTTGACGCTG;

GAPDH antisense primer, GCCAAATTCGTTGTCATACCAGGAA;

α-SMA sense primer, AATCCTGTGAAGCAGCTCCAG;

α-SMA antisense primer, CCCCTGATGTCTGGGACGT;

COLI a1 sense primer, AAGAATGGAGATGATGGGGAAG;

COLI a1 antisense primer, CTTAGGACCAGCAGGACCAG;

CRP2 sense primer, AGCCCAGCCTCGCTAGCTC;

CRP2 antisense primer, CAGAGAAAAGCAGCAGCGGTG;

CRP1 sense primer, AATGCCGAACTGGGGAGGAG;

CRP1 antisense primer, CACAGTGGTACTGTCCAGATTC;

TGF-β2 sense primer, GCCCTCCTACAGACTGGAGTCA;

TGF-β2 antisense primer, GAAGGCAGCAATTATCCTGCAC;

TGF-β1 sense primer, TGGAAGTGGATCCACGAGC;

TGF-β1 antisense primer, TCAGCTGCACTTGCAGGAG;

TGF-β3 sense primer, CTAAGCGGAATGAGCAGAGGATC;

TGF-β3 antisense primer, ATGCTCGTGGAATGTACAGTGC;

MMP-9 sense primer, CAAATGTGGGTGTACACAGGC;

MMP-9 antisense primer, TCGTAGGTCACGTAGCCCAC;

GATA4 sense primer, GTCCTGGGCCGCAGCCGC;

GATA4 antisense primer, TGACCCGTCCCATCTCGCC;

GATA5 sense primer, TCCTGGACTGCCGGGCCC;

GATA5 antisense primer, ATTGCACAGGTAGTGGCCGG;

GATA6 sense primer, GACGCCTGCCTGGCCCGC;

GATA6 antisense primer, CGGTGCCGTCCCGCCGC;

CRP2BP sense primer, TTCAGGTGGAAAGAAGATATCTG;

CRP2BP antisense primer, TTCCACCATCCTGGCTCTCC;

β-actin sense primer, GTCCACACCCGCCGCCAG;

β-actin antisense primer, TGGTGCCTGGGGCGCCCCAC;

γ-actin sense primer, GTCCACACCCGCCGCCAG;

γ-actin antisense primer, TCGTACTCCTGCTTGCTAATCC;

SRF sense primer, CAGCTTCACCCTCATGCCTG;

SRF antisense primer, ATGGTGGCGGGCAGCGTC;

MRTF-A sense primer, GTCAGGATGCACATTTTGGAAG;

MRTF-A antisense primer, TTTGGGATAGTTCACCTGGCC;

MRTF-B sense primer, ACCGAGGATGAAGTGGGACC;

MRTF-B antisense primer, GTTGCAGCCTCAGCTGGAGC.

**Molecular dynamics (MD) simulation**

The 3D structure of CRP2 (PDB ID 1qli) was used as an initial structure for the simulation of wild-type CRP2 (WT). The 3D structure of WT was used as a template model to build the homology models of mutant CRP2s.

Simulation system preparation was carried out using the leap module of the AMBER16 (Case et al., 2017) software package. The AMBER14SB (James et al., 2015) force field was applied for the corresponding proteins and the Zinc AMBER force field (Martin et al., 2010) (ZAFF) for zinc atoms and residues forming zinc finger structure. Then, the system was solvated in a TIP3P (Jorgensen et al., 1983) water box that extends at least 10.0A from the protein surface.

MD simulation was performed by pmemd. cuda module of AMBER16 (Case et al., 2017) software package. The systems were initially energy minimized using the steepest descent and conjugate gradient minimization algorithm. Each relaxed system was heated from 0K to 300K in a constant volume (NVT) ensemble over 20 ps. Then, systems were equilibrated in constant pressure (NPT) ensemble for 980 ps.

After the above three phases, the production phase MD simulation was performed over 300 ns in constant pressure (NPT) ensemble. The Particle mesh Ewald summation (PME) was employed to calculate electrostatic interaction by setting the cutoff at 12.0 Å for long-range interactions. The SHAKE algorithm was applied for all hydrogen-containing covalent bonds. Periodic boundary conditions were applied for all the systems. The simulation time step was set to 1.0 fs.

**References**

Case, D.A., Cerutti, D.S., Cheatham, III, T.E., Darden, T.A., Duke, R.E., Giese, T.J., Gohlke, H., Goetz, A.W., Greene, D., Homeyer, N., Izadi, S., Kovalenko, A., Lee, T.S., LeGrand, S., Li, P., Lin, C., Liu, J., Luchko, T., Luo, R., Mermelstein, D., Merz, K.M., Monard, G., Nguyen, H., Omelyan, I., Onufriev, A., Pan, F., Qi, R., Roe, D.R., Roitberg, A., Sagui, C., Simmerling, C. L., Botello-Smith, W.M., Swails, J., Walker, R.C., Wang, J., Wolf, R.M., Wu, X., Xiao, L., York, D.M., and Kollman. P.A. 2017. *AMBER 2017*, University of California, San Francisco.

James, A.M, Carmenza, M., Koushik, K., Lauren, W., Kevin, E.H., and Carlos, S. 2015. ff14SB: Improving the Accuracy of Protein Side Chain and Backbone Parameters from ff99SB. *J. Chem. Theory Comput.* 11, 3696–3713.

Jorgensen, W.L., Chandrasekhar, J., Madura, J.D., Impey, R.W., and Klein, M.L. 1983. Comparison of Simple Potential Functions for Simulating Liquid Water. *J.* *Chem. Phys.* 79, 926–935.

Martin, B.P., Yue, Y., Bing, W., László, F-M., Michael, N.W., and Kenneth, M.M. Jr. 2010. Structural Survey of Zinc-Containing Proteins and Development of the Zinc AMBER Force Field (ZAFF), *J. Chem. Theory Comput.* 6, 2935-2947.

**
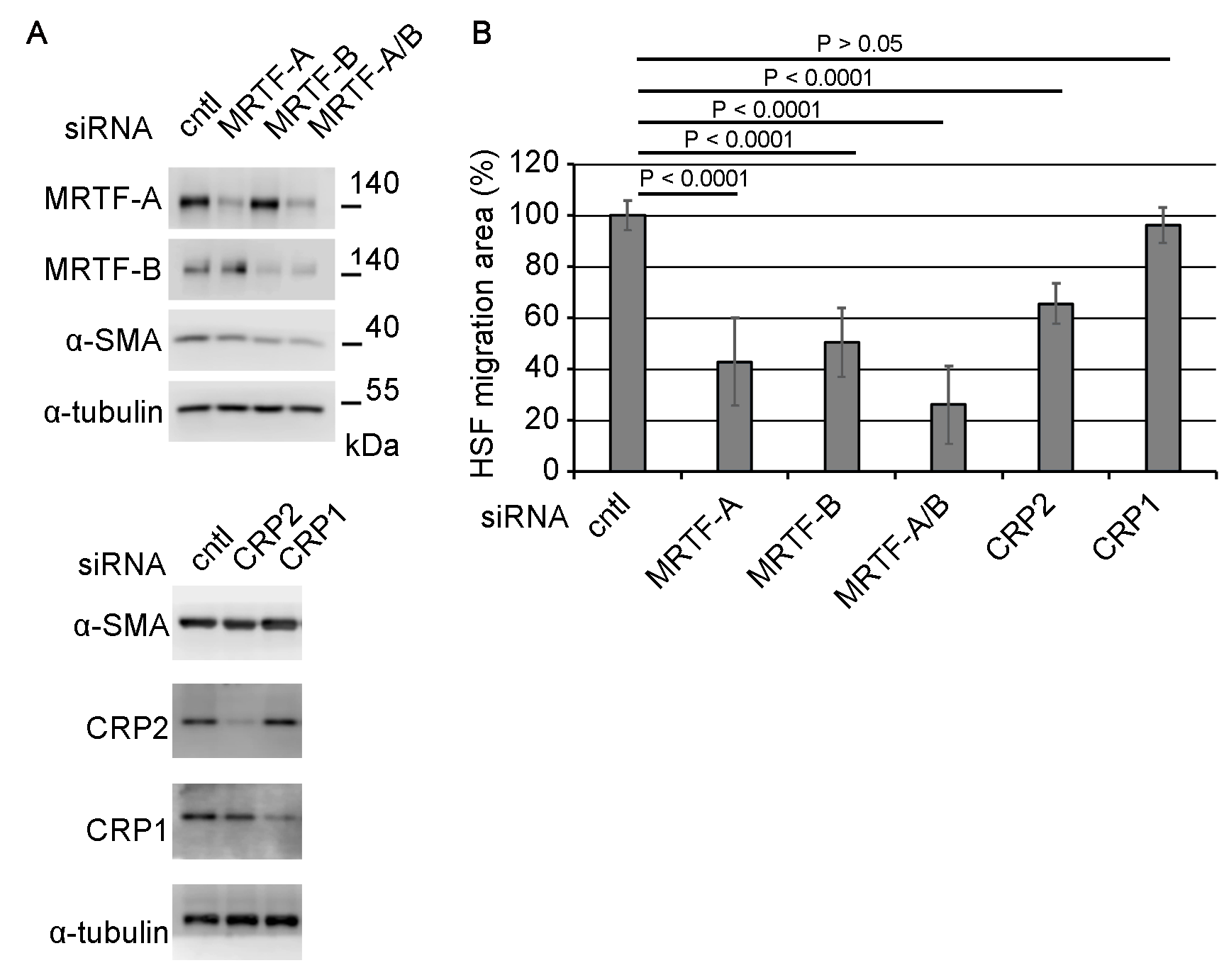
**

**Figure S1. Effects of KD with different siRNAs against MRTF, CRP1, and CRP2.** IB analysis **(A)** and wound healing assay **(B)** represent their effects on the expression of the indicated proteins and cell motility (see the legend of Fig. 1). ANOVA shows a significant difference in the wound healing assay (p < 0.0001).

**
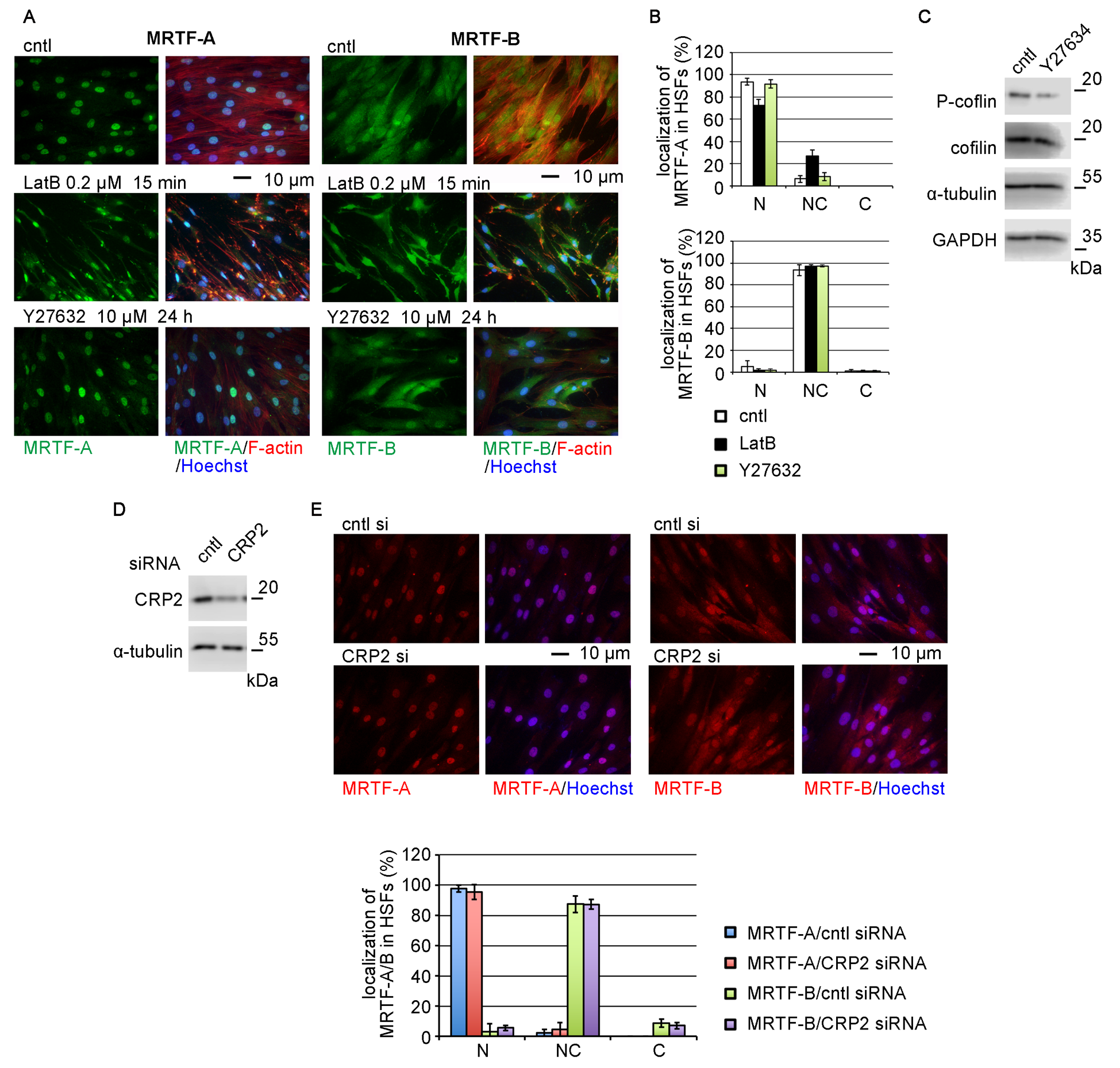
**

**Figure S2. Subcellular localization of MRTF-A/B in HSFs.**

**(A-C)** Effect of LatB or Y27632 on the subcellular localization of MRTF-A and MRTF-B in HFSs. HSEs were cultured under serum-stimulated conditions for 2 days. For the last indicated periods, HSFs were treated with either vehicle (DMSO) (control [cntl]), 10 μM Y27632, or 0.2 μM LatB. Cells were stained with anti-MRTF-A or anti-MRTF-B antibody (green), phalloidine-Alexa 568 (red), and Hoechst 33258 (blue) **(A)**. These are representative images from at least three independent experiments. Images were analyzed as described in Materials and Methods **(B)**. Subcellular localization of MRTFs is categorized into three groups: nuclear-specific localization (N); diffuse distribution in the nucleus and the cytoplasm (NC); and cytoplasmic localization (C). The statistical analyses are as follows: ANOVA between control and LatB, P < 0.01; ANOVA between control and Y27634, P = 0.6061 for MRTF-A. The effects of these agents on the subcellular localization of MRTF-B are less significant (ANOVA P = 0.6963). Multiple comparisons of the effect of LatB on the subcellular localization of MRTF-A as follows: pair N-C, P < 0.0001; pair N-NC, P < 0.0001; pair NC-C, p = 0.0019. IB analysis with whole-cell lysates to detect the phosphorylation state of cofilin **(C)**. **(D and E)** Effect of siRNA-mediated KD of CRP2 on the subcellular localization of MRTF-A and MRTF-B in HSFs. HSFs were transfected with the indicated siRNAs and cultured for 2 days. IB analysis with whole-cell lysates to detect the expression level of CRP2 **(D)**. Cells were stained with anti-MRTF-A antibody or anti-MRTF-B (red) and Hoechst 33258 (blue) **(E)**. These are representative images. The lower graph shows the quantitative data. ANOVA shows no significant difference in the effects of each siRNA on the subcellular localization of MRTF-A and MRTF-B (P = 1.000).


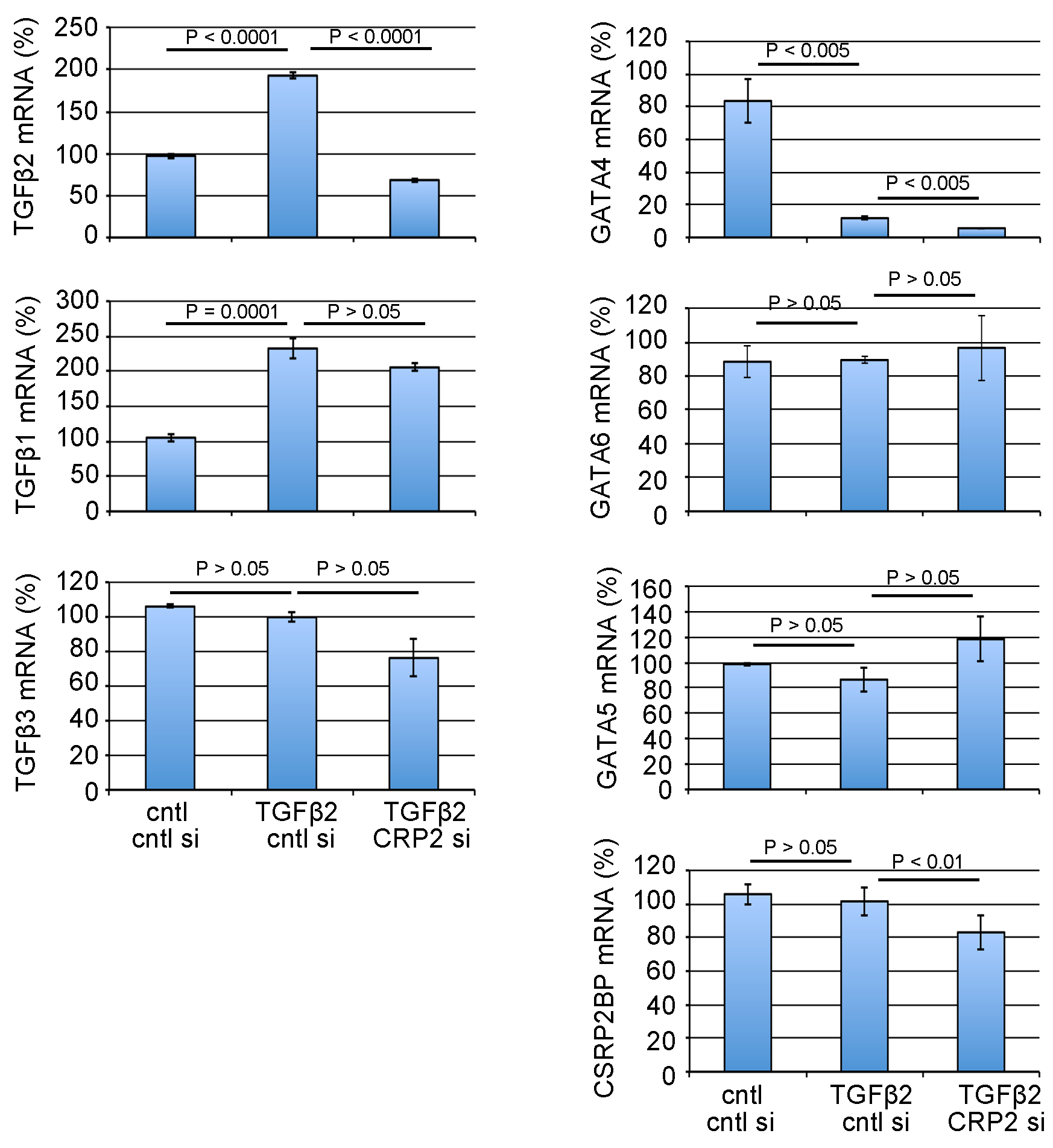


**Figure S3. Effect of CRP2 KD on the expression of TGF-βs, GATA family members, and CRP2BP in TGF-β2-stimulated HSFs.**

HSFs were transfected with the indicated siRNA (si) and then cultured for 2 days. For the last 24 hours, HSFs were cultured with either vehicle (PBS containing 0.1% BSA) (control [cntl]) or 2 ng/ml TGF-β2 (see the legend of Fig. 2). RT-qPCR quantified the expression levels of the indicated mRNAs. Their expression levels in control (cntl) siRNA-transfected HSFs cultured under non-stimulated conditions were set at 100% (means ± SEMs of three independent experiments). ANOVA shows a significant difference in the RT-qPCR analysis of TGF-β1, TGF-β2, TGF-β3, GATA4, and CRP2BP (p < 0.01) but not in others (P > 0.05).

**
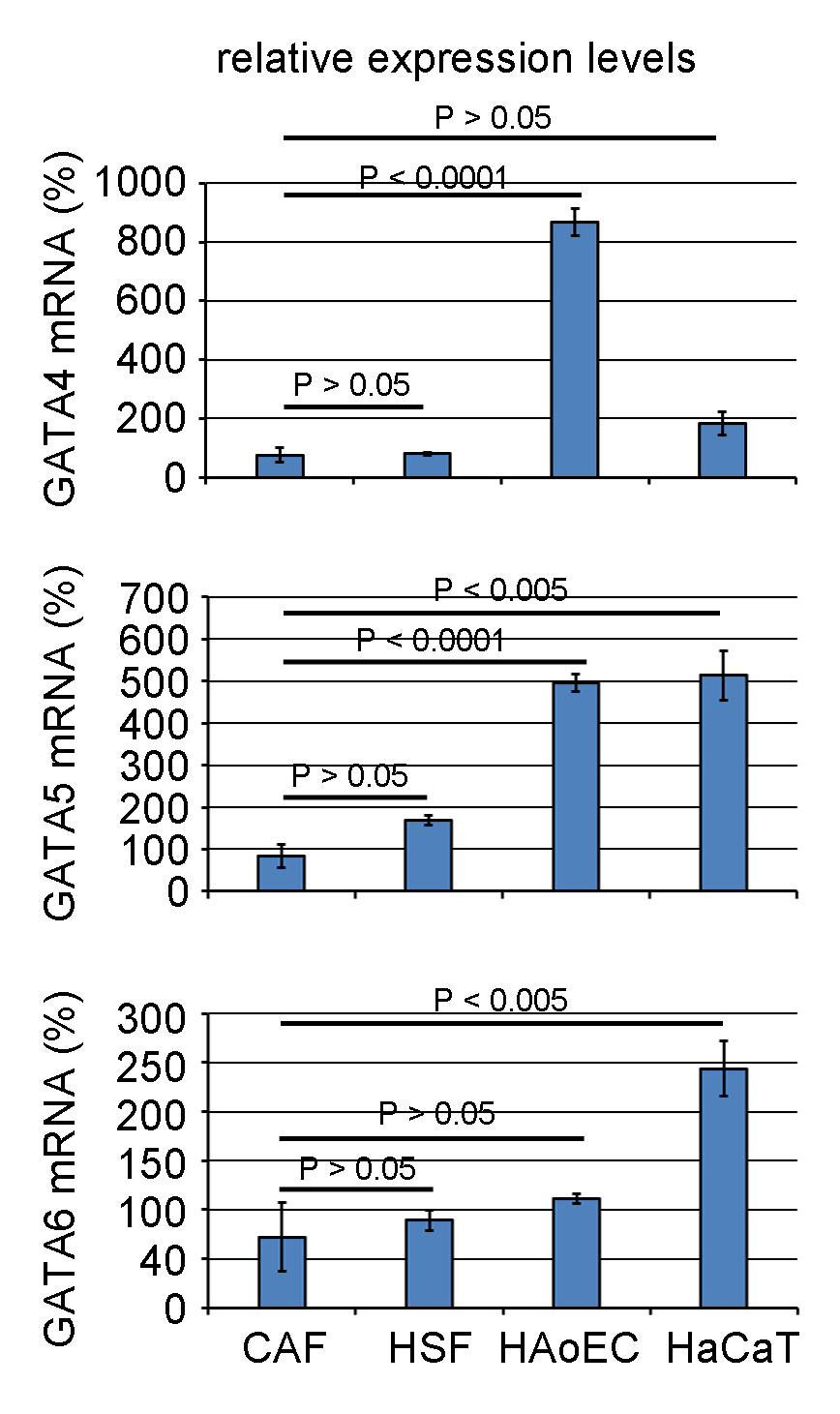
**

**Figure S4. Comparison of the expression of GATA family members in CAFs, HSFs, HAoECs, and HaCaT cells.**

RT-qPCR quantified the expression levels of the indicated mRNAs in CAFs, HSFs, HAoECs, and HaCaT cells. Their expression levels in CAFs were set at 100% (means ± SEMs of three independent experiments). ANOVA shows a significant difference in the RT-qPCR analysis of each GATA family member (p < 0.001).

**
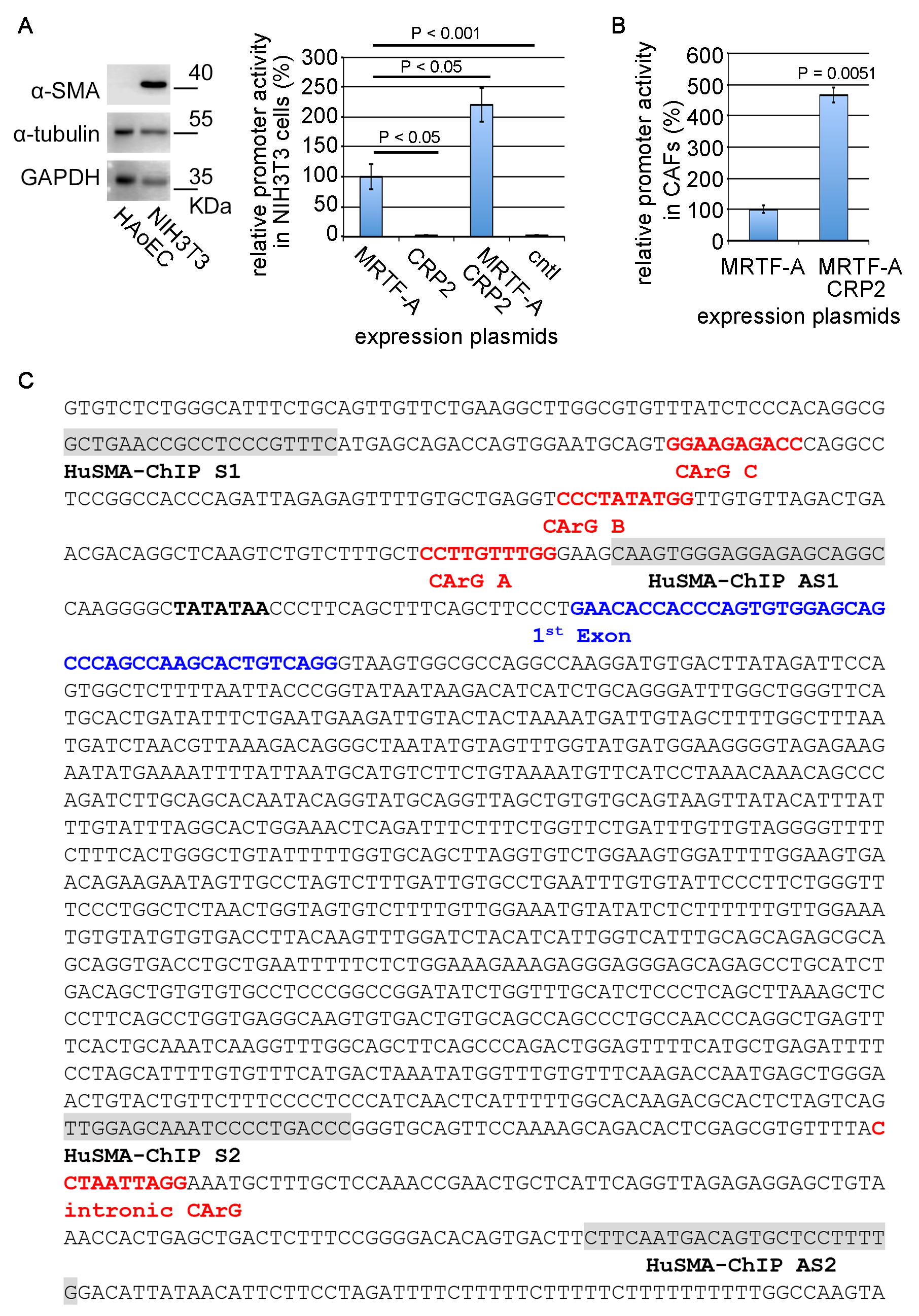
**

**Figure S5. Promoter assays in NIH3T3 cells and CAFs and the genomic sequence**

**of human α-SMA gene expanding from the 5’ non-coding region to the first intron. (A and B)** Promoter assays in NIH3T3 cells **(A)** and CAFs **(B)**. IB analysis with whole-cell lysates from HAoECs and NIH3T3 cells (**A**). NIH3T3 cells or CAFs were transfected with 3xCArG-Luciferase report plasmid, pSVβ−gal, and the indicated expression plasmids (see the legend of Fig. 5A). ANOVA shows a significant difference in the promoter assay (P < 0.0001). The luciferase activities induced by exogenous Flag-MRTF-A alone were set at 100%. **(C)** Genomic sequence of human α-SMA gene expanding from the 5’ non-coding region to the first intron (Ensembl genome browser, ACTA2 [ENSG00000107796]). Red and blue letters indicate the four functional CArG-boxes (GArGs A to C and intronic CArG) and the sequence of the first exon, respectively. The shaded sequences are the primers for ChIP-PCR. They are as follows: HuSMA-ChIP S1 and HuSMA-ChIP AS1 for the three CArG-boxes in the promoter region; HuSMA-ChIP S2 and HuSMA-ChIP AS2 for the intronic CArG-box.

**
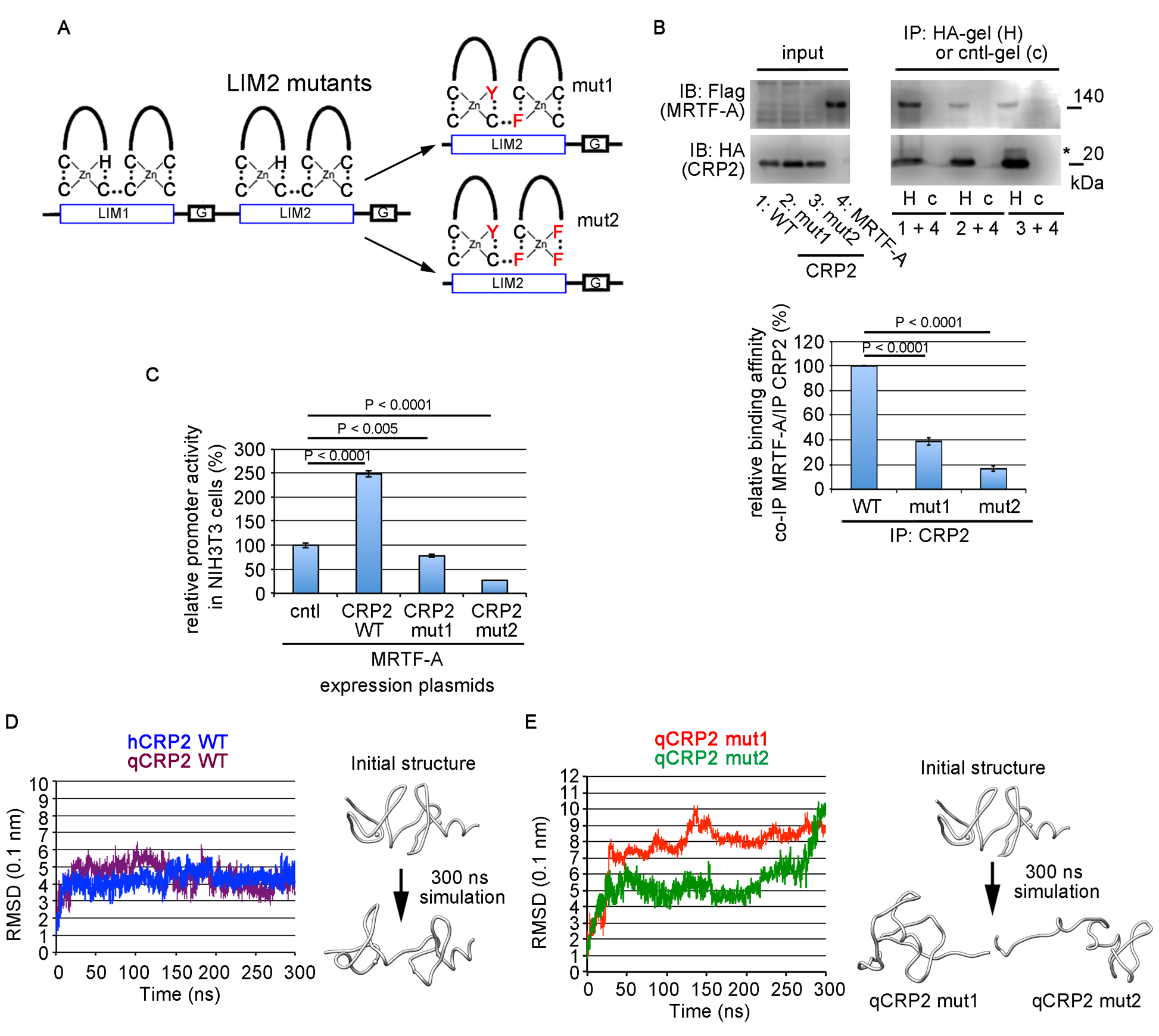
**

**Figure S6. Significance of the 3D structure of the LIM domain in the binding of CRP2 to MRTF-A.**

**(A)** Overview of the C-terminal LIM domain (LIM2) mutants of CRP2. LIM1 and LIM2, LIM domain; G, glycine-rich domain. **(B)** Binding affinities of human CRP2 mutants (mut1 and mut2) to MRTF-A. In vitro protein binding assay was performed as described in the legends of Figs. 5 and 6. In brief, mixtures of the indicated in vitro translated proteins (proteins numbered in the input) were subjected to IP/IB analysis. The graph shows the quantification of their binding affinities. The binding affinity of wild-type CRP2 (WT) was set at 100% (means ± SEMs of three independent experiments). ANOVA shows a significant difference in the binding assay (P < 0.0001). Asterisk (*) indicates the IgG light chain. **(C)** Synergistic effects of MRTF-A and each of the indicated CRP2 mutants on the SRF/CArG-box-mediated transcription. Promoter assay in NIH3T3. Cells were transfected with 3xCArG-Luciferase report plasmid, pSVβ−gal, and the indicated expression plasmids (see the legend of Fig. 5A). The promoter activity induced by exogenous MRTF-A alone was set at 100%. ANOVA shows a significant difference in the promoter assay (P < 0.0001). **(D)** MD simulations of the C-terminal half of quail wild-type (WT) and the mutant CRP2 proteins (mut [quail/human] = human CRP2 WT). In this mutant CRP2, the variant sequence between quail and human CRP2 proteins is converted to the human CRP2 sequence. The graph and schematic diagram show their MD simulations. The images show the initial and final structures of human CRP2 WT. These are representative results from two independent simulations. **(E)** MD simulations of the C-terminal half of mutant quail CRP2 proteins corresponding to the human mutants [mut1 and mut2 **(A)**].

**
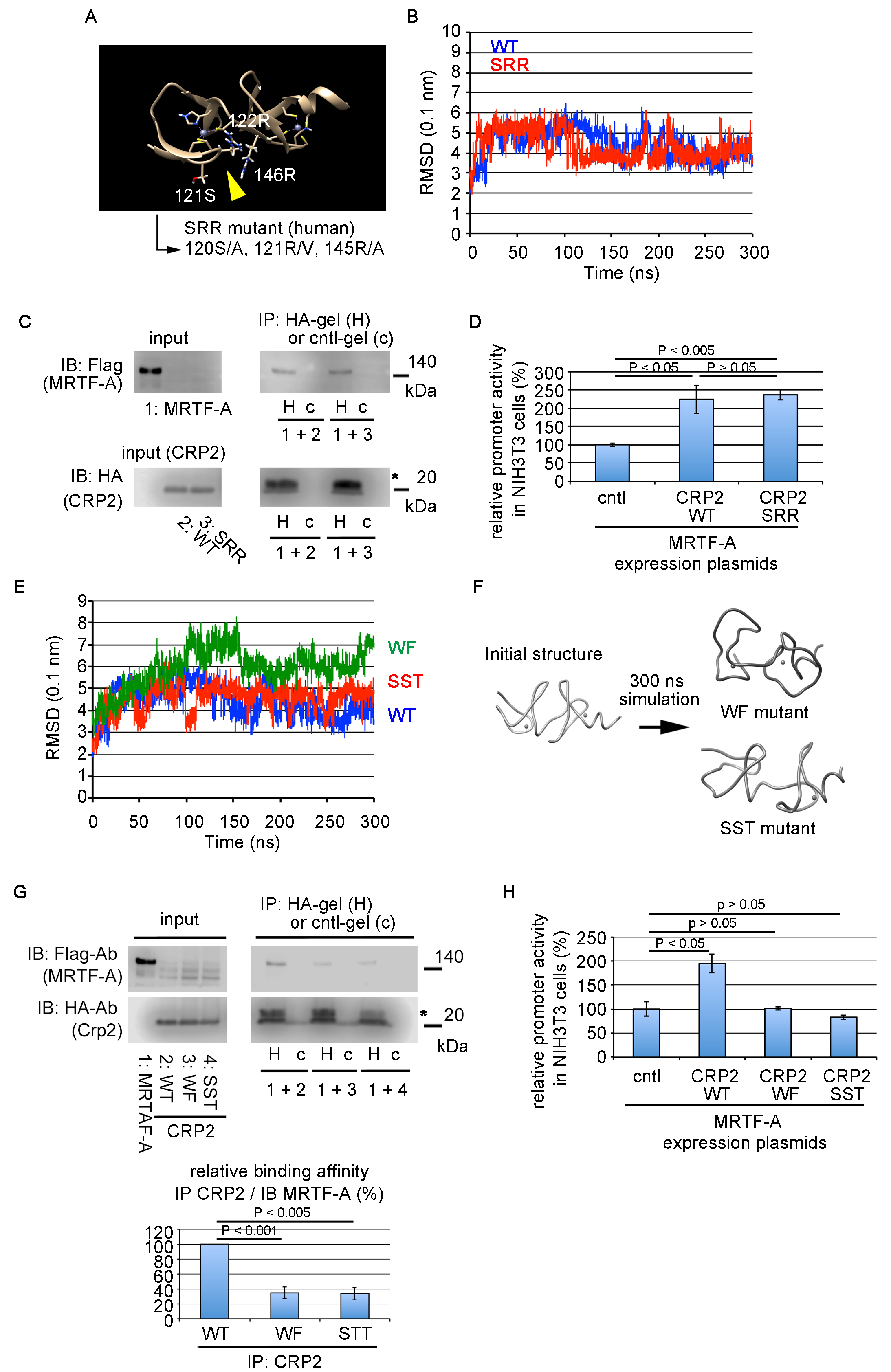
**

**Figure S7. 3D structural and functional characterization of three CRP2 mutants (SRR, WF, SST).**

**(A)** A yellow arrowhead indicates the largest protein-binding cavity (see Fig. 7B). The 3D positions of three polar amino acids, 121S, 122R, and 146R in quail CRP2 (120S, 121R, and 145R in human CRP2) [yellow shading letters in Fig. 7A]. **(B)** MD simulations of the C-terminal half of quail wild-type CRP2 (WT) and the CRP2 SRR mutant proteins. The graph shows their MD simulations. They are representative results from two independent simulations. **(C and D)** Examination of the biological functions of the CRP2 SRR mutant. Comparison of the binding affinities between wild-type (WT) CRP2 and the CRP2 SST mutant to MRTF-A by in vitro protein binding assay **(C)**. Mixtures of the indicated in vitro translated proteins (proteins numbered in the input panels) were subjected to IP/IB analysis. Synergistic effect of MRTF-A and the CRP2 SRR mutant on the SRF/CArG-box-mediated transcription **(D)**. Promoter assay in NIH3T3. Cells were transfected with 3xCArG-Luciferase report plasmid, pSVβ−gal, and the indicated expression plasmids (see the legend of Fig. 5A). The promoter activity induced by exogenous MRTF-A alone was set at 100%. ANOVA shows a significant difference in the promoter assay (P = 0.0019).  **(E and F)** MD simulation of the C-terminal half of quail wild-type CRP2 (WT), the CRP2 WF mutant, and the CRP2 SST mutant proteins. Each mutant sequence is shown in the alignment of the CRP2 sequence (Fig. 7A): red shading letters for the SST mutant and green shading letters for the WF mutant. Graph **(E)** and schematic diagram **(F)** show their MD simulations. They are representative results from two independent simulations. **(G and H)** Examination of the biological functions of these mutant CRP2 proteins. Comparison of the binding affinities to MRTF-A among wild-type CRP2 (WT), the CRP2 SST mutant, and the CRP2 WF mutant by in vitro protein binding assays **(G)**. Mixtures of the indicated in vitro translated proteins (proteins numbered in the input panels) were subjected to IP/IB analysis. The graph shows quantitative results. Synergistic effect of MRTF-A and each of the indicated CRP2 mutants on the SRF/CArG-box-mediated transcription **(H)**. Promoter assay in NIH3T3 (see above description **(D)**). ANOVA shows a significant difference in both assays **(G and H)** (P < 0.0001). Asterisks (*) indicate the IgG light chain (**C** and **G**).

**Legends for supplemental videos (Videos 1 - 6)**

**Video 1. MD simulation of wild-type CRP2.**

MD simulation of the C-terminal half of wild-type quail CRP2 (82-194 amino acids). The movie corresponds to Fig. 7C, D, Fig. S6D, and Fig. S7B, E, F.

**Video 2. MD simulation of CRP2 SRR mutant.**

MD simulation of the C-terminal half of mutant quail CRP2 [SRR mutant] (82-194 amino acids). The movie corresponds to Fig. S7B.

**Video 3. MD simulation of CRP2 LL mutant.**

MD simulation of the C-terminal half of mutant quail CRP2 [LL mutant] (82-194 amino acids). The movie corresponds to Fig. 7C, D.

**Video 4. MD simulation of CRP2 ETT mutant.**

MD simulation of the C-terminal half of mutant quail CRP2 [ETT mutant] (82-194 amino acids). The movie corresponds to Fig. 7C, D.

**Video 5. MD simulation of CRP2 SST mutant.**

MD simulation of the C-terminal half of mutant quail CRP2 [SST mutant] (82-194 amino acids). The movie corresponds to Fig. S7E, F.

**Video 6. MD simulation of CRP2 WF mutant.**

MD simulation of the C-terminal half of mutant quail CRP2 [WF mutant] (82-194 amino acids). The movie corresponds to Fig. S7E, F.
